# Ras-mutant cancers are sensitive to small molecule inhibition of V-type ATPases in mice

**DOI:** 10.1038/s41587-022-01386-z

**Published:** 2022-07-25

**Authors:** Bhairavi Tolani, Anna Celli, Yanmin Yao, Yong Zi Tan, Richard Fetter, Christina R. Liem, Adam J. de Smith, Thamiya Vasanthakumar, Paola Bisignano, Adam D. Cotton, Ian B. Seiple, John L. Rubinstein, Marco Jost, Jonathan S. Weissman

**Affiliations:** 1 Thoracic Oncology Program, Department of Surgery, Helen Diller Family Comprehensive Cancer Center, University of California, San Francisco, CA, USA; 2 Laboratory for Cell Analysis Core Facility, Helen Diller Family Comprehensive Cancer Center, University of California, San Francisco, CA, USA; 3 Department of Pharmaceutical Chemistry and Cardiovascular Research Institute, University of California, San Francisco, CA, USA; 4 Molecular Medicine Program, The Hospital for Sick Children, Toronto, ON, Canada; 5 Howard Hughes Medical Institute, Department of Biology, Stanford University, Stanford, CA, USA; 6 Department of Cellular and Molecular Pharmacology, University of California, San Francisco, San Francisco, CA, USA; 7 Center for Genetic Epidemiology, Department of Population and Public Health Sciences, Keck School of Medicine of University of Southern California, Los Angeles, CA, USA; 8 Department of Biochemistry, The University of Toronto, Toronto, ON, Canada; 9 Department of Medical Biophysics, The University of Toronto, Toronto, ON, Canada; 10 Department of Microbiology & Immunology, University of California, San Francisco, CA, USA; 11 Department of Microbiology, Harvard Medical School, Boston, MA, USA; 12 Howard Hughes Medical Institute, Massachusetts Institute of Technology, Cambridge, MA, USA; 13 Whitehead Institute for Biomedical Research, Massachusetts Institute of Technology, Cambridge, MA, USA; 14 David H. Koch Institute for Integrative Cancer Research, Massachusetts Institute of Technology, Cambridge, MA, USA; 15 Department of Biology, Massachusetts Institute of Technology, Cambridge, MA, USA

## Abstract

Mutations in Ras family proteins are implicated in 33% of human cancers, but direct pharmacological inhibition of Ras mutants remains challenging. As an alternative to direct inhibition, we screened for sensitivities in RAS-mutant cells and discovered 249C as a Ras-mutant selective cytotoxic agent with nanomolar potency against a spectrum of RAS-mutant cancers. 249C binds to vacuolar (V)-ATPase with nanomolar affinity and inhibits its activity, preventing lysosomal acidification and inhibiting autophagy and macropinocytosis pathways that several Ras-driven cancers rely on for survival. Unexpectedly, potency of 249C varies with the identity of the RAS driver mutation, with the highest potency for *KRAS*G13D and G12V both *in vitro* and *in vivo,* highlighting a mutant-specific dependence on macropinocytosis and lysosomal pH. Indeed, 249C potently inhibits tumor growth without adverse side effects in mouse xenografts of *KRAS*-driven lung and colon cancers. A comparison of isogenic SW48 xenografts with different *KRAS* mutations confirmed that *KRAS*G13D/+ (followed by G12V/+) mutations are especially sensitive to 249C treatment. These data establish proof-of-concept for targeting V-ATPase in cancers driven by specific *KRAS* mutations such as *KRAS*G13D and G12V.

## Introduction

Mutations that constitutively activate members of the Ras family of oncogenes (H-Ras, N-Ras, and K-Ras) are collectively responsible for about one third of all human cancers; *KRAS* mutations in particular are implicated in the most fatal malignancies: pancreatic (91%), colon (42%) and lung (33%)^1–4^. Despite decades-long efforts, we still lack clinically-approved drugs for the majority of oncogenic *KRAS* variants (apart from Amgen’s *KRAS*G12C inhibitor), in large part due to substantial challenges in directly inhibiting mutant *KRAS*. As an alternative strategy for pharmacological intervention, efforts to map potentially druggable neomorphic dependencies of *KRAS* mutant cancers have identified, among others, metabolic adaptations concomitant with activating *RAS* mutations, including high levels of basal autophagy, a phenomenon termed “autophagy addiction”^5–9^, and constitutive activation of macropinocytosis (MP)^10^. Both autophagy and MP provide nutrients to promote tumor growth by degrading macromolecules in acidic lysosomes produced by vacuolar H^+^ ATPases (V-ATPases)^11,12^, and V-ATPase has been reported as an essential regulator of *RAS*-induced MP for nutrient supply, suggesting that targeting V-ATPase could be exploited to curtail the metabolic adaptation of *RAS*-mutant cells^10^.

V-ATPase is a multi-subunit transmembrane complex that operates as an ATP-driven rotary proton pump, coupling ATP hydrolysis in its peripheral V_1_ domain to proton translocation from the cytoplasm to the lumen of organelles through its membrane integral Vo domain. Through this activity, V-ATPase is essential for lysosome acidification and thus lysosomal degradation^13,14^ and more broadly regulates a wide array of membrane trafficking and intracellular transport processes^15^. Although specific V-ATPase inhibitors such as Bafilomycin A1 (BafA1) have been developed and serve as important tool compounds, their clinical use is limited by toxicity^16^. Additional autophagy-modulating agents have been described, such as the repurposed antimalarial compound chloroquine (CQ) and its less toxic derivative hydroxychloroquine (HCQ), which impair lysosomal acidification by targeting Palmitoyl-protein thioesterase 1 (PPT1), but these compounds have severe side effects by interfering with human *ether-à-go-go*-related gene (hERG) responsible for electrical activity of the heart^17,18^ and they can also cause gastrointestinal side effects, impact visual and auditory function, and lead to hypoglycemia. In over 60 clinical trials (completed or ongoing), the use of CQ or HCQ alone as a monotherapy do not demonstrate significant therapeutic efficacy^19^. Although second-generation analogs of HCQ such as ROC325, Lys05, DC661, and DQ661 show enhanced preclinical lysosomal-autophagic inhibition and antitumor activity as single agents *in vitro* and *in vivo*, it remains to be determined whether any of them will show efficacy in clinical investigations while avoiding unacceptable toxicities^17,20^. These limitations motivate an urgent need to identify and develop additional autophagy/MP inhibitors with clean safety profiles.

Here, we report the discovery and characterization of a class of dihydro-pyrazole-5-carboxamide compounds with exquisite potency in killing *KRAS* mutant cells and excellent pharmacological properties. By combining chemoproteomics, comparative profiling, and chemo-genetic CRISPR screening, we determined that our lead compound 249C binds to and inhibits V-ATPase, resulting in inhibition of autophagy, MP, and lysosomal pH. Through this activity, 249C selectively kills *KRAS/BRAF* mutant cells, with the highest activity against *KRAS*G13D and G12V mutant cells, pointing to excessive reliance on V-ATPase as a unique vulnerability resulting from these *KRAS* mutations. For ~1 year, the molecule has been in a multi-center Phase Ia/b dose escalation and dose expansion clinical trial for the following indications: RAS mutations, lung, colon, and pancreatic cancers across 5 different sites in the U.S. (NCT04678648 | 249C = RSC1255).

## Results

### 249C is a potent inhibitor of Ras- and Raf-mutant cells

**Figure 1:**
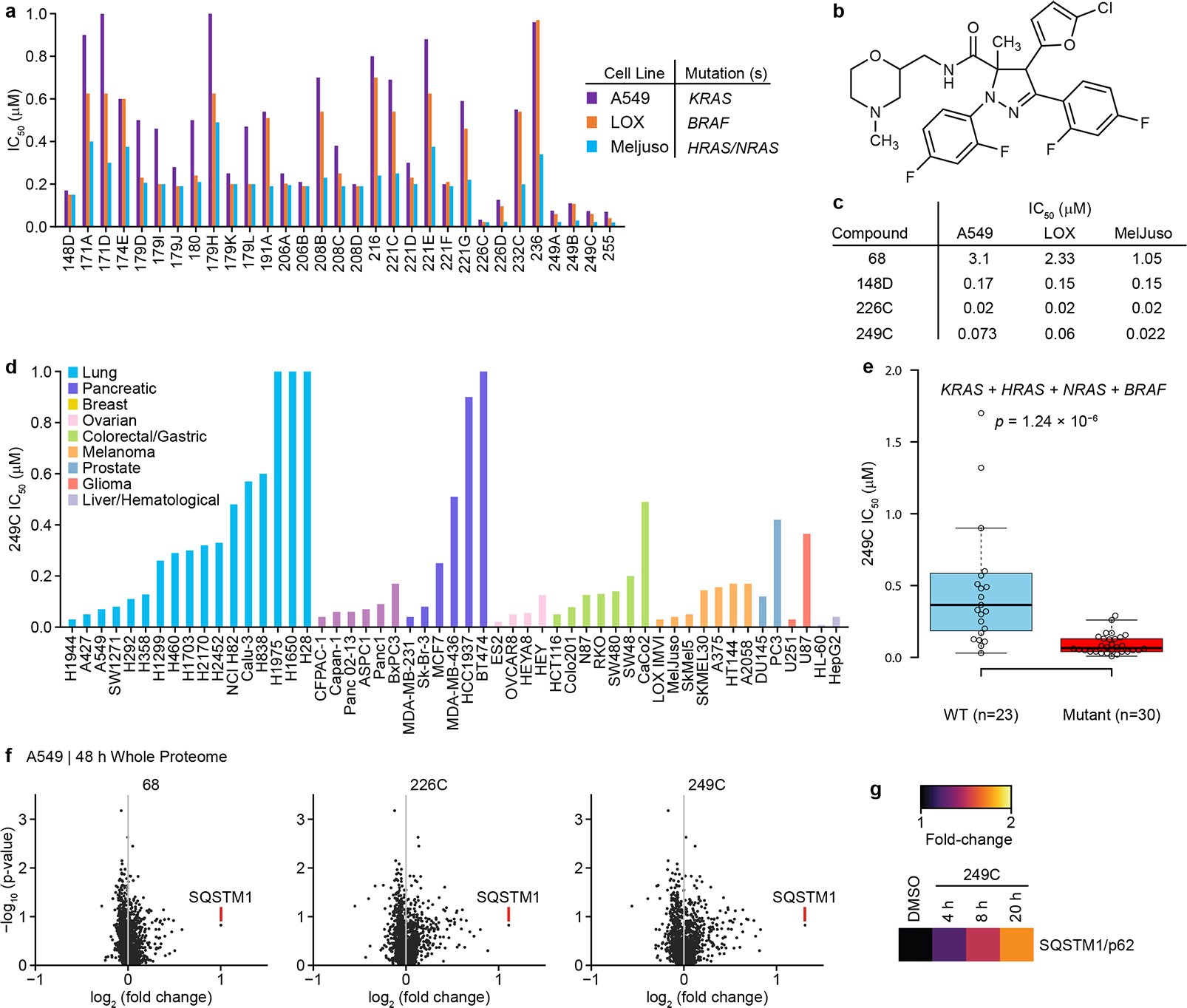
Optimization of Potency and Drug Properties of Small Molecule Inhibitors of *Ras*- and *Raf*-mutant Cells using Viability Screens. (**A**) Cell viability screen of ~300 small molecules in A549 (*KRAS*G12S), LOX IMVI (*BRAF*V600E), and MelJuso (*HRAS*G13D and *NRAS*Q61L). Cells were treated with vehicle DMSO or increasing concentrations of small molecules in triplicate for 72 h and ATP content/viability was measured using CellTiter-Glo Luminescent Cell Viability Assay. The results shown for all cell viability figures are representative of at least two independent experiments. The top 30 compounds (p<0.05 compared to vehicle-treated controls, two-sided *t*-test) with the lowest IC_50s_ are presented. n=3 (**B**) Structure of dihydro-pyrazole-5-carboxamide small molecule hit 249C that shows inhibition of viability in both *Ras* and *Raf* mutant cells. (**C**) Evolution of the Structure Activity Relationship (SAR) and IC_50_s of the first (68) and last (249C | lead due to drug-like properties) compounds in the screen. (**D**) 249C sensitivity for a panel of 53 cancer cell lines (including lung, pancreatic, breast, ovarian, hematological/liver, prostate, colorectal cancers, glioma and melanoma). (**E**) IC_50_ values stratified by the mutation status of *KRAS, HRAS, NRAS,* and *BRAF* (two-sided Wilcoxon rank sum test | *p* = 1.24 × 10^−6^ | See also Fig.S1). The center line represents the median, the upper and lower bounds of the box indicate the interquartile range (IQR, the range between the 25th and 75th percentiles), and whiskers extend to the highest and lowest values within 1.5 times the IQR. (**F**) Quantitative whole proteome analysis of the effects of small molecule treatment by mass spectrometry. A549 cells were treated with DMSO or 68 (30 μM), 226C (1μM) or 249C (1μM) for 48 h prior to analysis of proteomes with TMT labeling followed by quantification relative to the DMSO control. Autophagy receptor, SQSTM1, was identified as highly up-regulated and common for our family of compounds by proteomic mass spectrometric screening in small molecule-treated A549 mutant *KRAS* cells. More data can be found in Fig.S2 and Supplementary Tables 1–3. (**G**) Quantitative whole proteome analysis of 249C-treated (1μM) A549 cells over time relative to DMSO control.

We had recently synthesized ~300 dihydro-pyrazole-5-carboxamide small molecules to target a developmental pathway (Fig.S1; Table S1). Here, to evaluate the ability of these molecules to also inhibit growth of *Ras* and *Raf* mutant cells, we screened these molecules for anti-proliferative activity against three human cancer cell lines with mutations in *Ras* or *Raf*: A549 (*KRAS*G12S), LOX-IMVI (*BRAF*V600E), and MelJuso (*HRAS*G13D and *NRAS*Q61L). Using structure-activity relationships, we observed that several compounds potently reduced viability of all 3 cell lines (Fig. 1a; Table S1–3). Simultaneous evaluation of drug-like properties and half-maximal inhibitory concentration (IC_50_) values revealed 249C as the lead compound based on physico-chemical properties (Fig. 1b–c). Compound 249C was chosen over 226C for further studies due to the presence of a tertiary amine, imparting higher water solubility, and a slightly lower cLogP (3.07 vs 3.33). To further evaluate determinants of sensitivity to 249C, we measured its potency against 53 cell lines (lung, pancreatic, breast, ovarian, colorectal/gastric, prostate, liver/hematological, glioma and melanoma) (Fig. 1d). Comparing IC_50_ values to gene mutation data (CCLE) revealed that the presence of at least one of *KRAS*, *HRAS*, *NRAS*, and/or *BRAF* mutations was significantly associated with lower IC_50_ values (*p*=1.24 × 10^−6^), with median IC_50_ values of 65 nM in *KRAS*/*HRAS*/*NRAS*/*BRAF* mutant cells versus 365 nM in cells with no mutations in these genes (Fig. 1e). *KRAS* showed the strongest single gene association with IC_50_ values (*p*=2.34 × 10^−3^) (Fig.S1b–c). We next mapped the response of A549 cells to these compounds using mass spectrometry (LC–MS/MS)-based protein profiling coupled with quantitative tandem mass tags labeling^21^. Upon treatment of cells with vehicle or molecules 68, 226C and 249C, we observed substantially elevated levels of the autophagy receptor SQSTM1/p62 common to this family of compounds (Fig. 1f) in a time-dependent fashion (Fig. 1g), suggesting that these compounds impact autophagy.

### 249C inhibits autophagy

**Figure 2.**
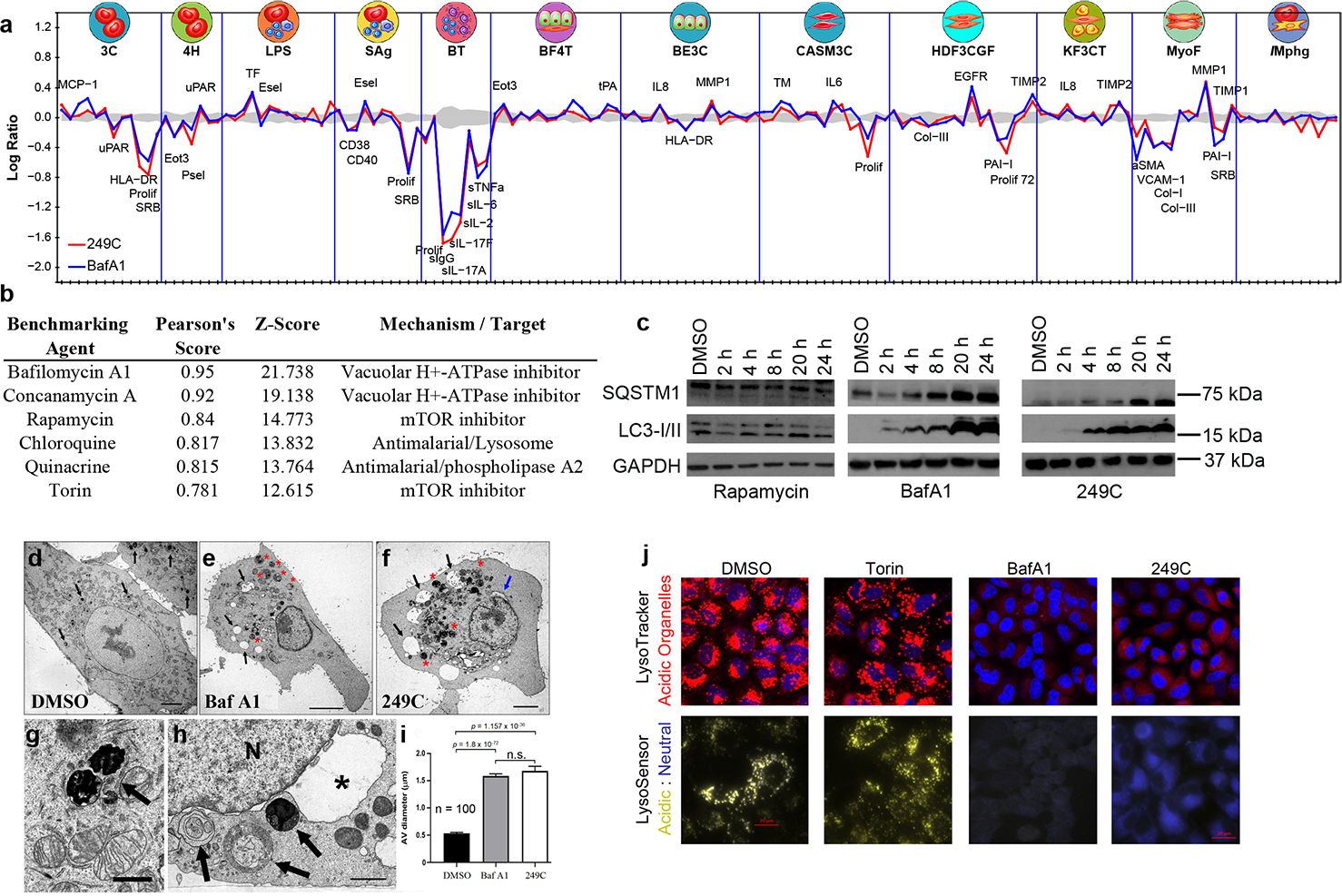
Lead Small Molecule 249C Phenocopies V-ATPase Inhibitor BafA1. (**A**) Overlay of 148 biomarker responses in a panel of 12 primary cell-based systems for **249C** and **BafA1**. BafA1 had the highest similarity out of 4000 molecules. (**B**) Pearson correlation and *z*-scores for observed phenotypes of 249C compared to known database drugs (see also Supplementary Table 4). (**C**) Treatment with 249C or the autophagy inhibitor BafA1 resulted in upregulation of autophagy markers SQSTM1/p62 and LC3-I/II over time but not with autophagy inducer, Rapamycin. (**D**) Representative electron micrographs of A549 cells treated with the DMSO vehicle, BafA1 (**E**) or 249C (**F**). Arrows in (D): very densely staining phagosomes/lysosomes. (**G**) shows two of these structures at higher magnification from a DMSO treated cell (arrow). Arrows in (E) and (F): large, clear vacuoles within the cells that are absent in (A); red asterisks in (E) and (F) large multivesicular autophagic vesicles (AVs). (**H**) Multivesicular AVs in a cell treated with 249C (arrows). The nucleus (N) in this cell exhibits a greatly distended portion of the nuclear envelope (asterisk) that resembles the large vacuoles seen in the cytoplasm of BafA1- and 249C-treated cells. (**I**) Quantification of mean ± SEM of the size of the multivesicular AVs from counts of DMSO -, BafA1 (*p* = 1.836 × 10^−72^)- and 249C-treated cells (*p* = 1.157 × 10^−36^). Scale bars in (D-F), 5 μm; (G-H), 1 μ m. Two-sided *t*-test, n=124 n.s. not significant. (**J**) Treatment with autophagy inhibitor, BafA1, and 249C decreased staining of acidic organelles (red | LysoTracker) and resulted in an increase in lysosomal pH (yellow: acidic; blue: neutral | LysoSensor) relative to the DMSO control and autophagy inducer, Torin1, in A549 *KRAS* mutant cells. Representative images from n >3.

To characterize the mode of action of our lead compound 249C, we used comparative profiling in a panel of primary cell-based systems designed to model disease biology^22^. Compared to 4,000 other drugs, the phenotypes elicited by 249C most closely resembled those elicited by the V-ATPase inhibitor BafA1 (Pearson *r* = 0.95 across all measurements, Fig. 2a, 2b, Fig.S2–3, Tables S4). 249C also exhibited similarity, albeit to a lesser degree, to the mTOR inhibitors Rapamycin and Torin1 and the antimalarials CQ and quinacrine. 249C and BafA1 elicited progressive accumulation of SQSTM1/p62 and LC3-II in A549 cells, consistent with a block in autophagic flux; by contrast, the autophagy inducer Rapamycin did not (Fig. 2c). Using transmission electron microscopy, we further observed late-stage autophagy inhibition and increased autophagic vesicle accumulation in A549 cells treated with 249C and BafA1 (Fig. 2d–i). Finally, treatment with both 249C and BafA1 caused a reduction in the presence of acidic organelles (red: Fig. 2j – top panel) and an increase in pH (yellow: acidic; blue: neutral | Fig. 2j – bottom panel) of A549 cells relative to DMSO and Torin1, confirming that 249C blocks lysosomal acidification and autophagy progression.

### Chemical–genetic screen reveals V-ATPase as the target of 249C

**Figure 3.**
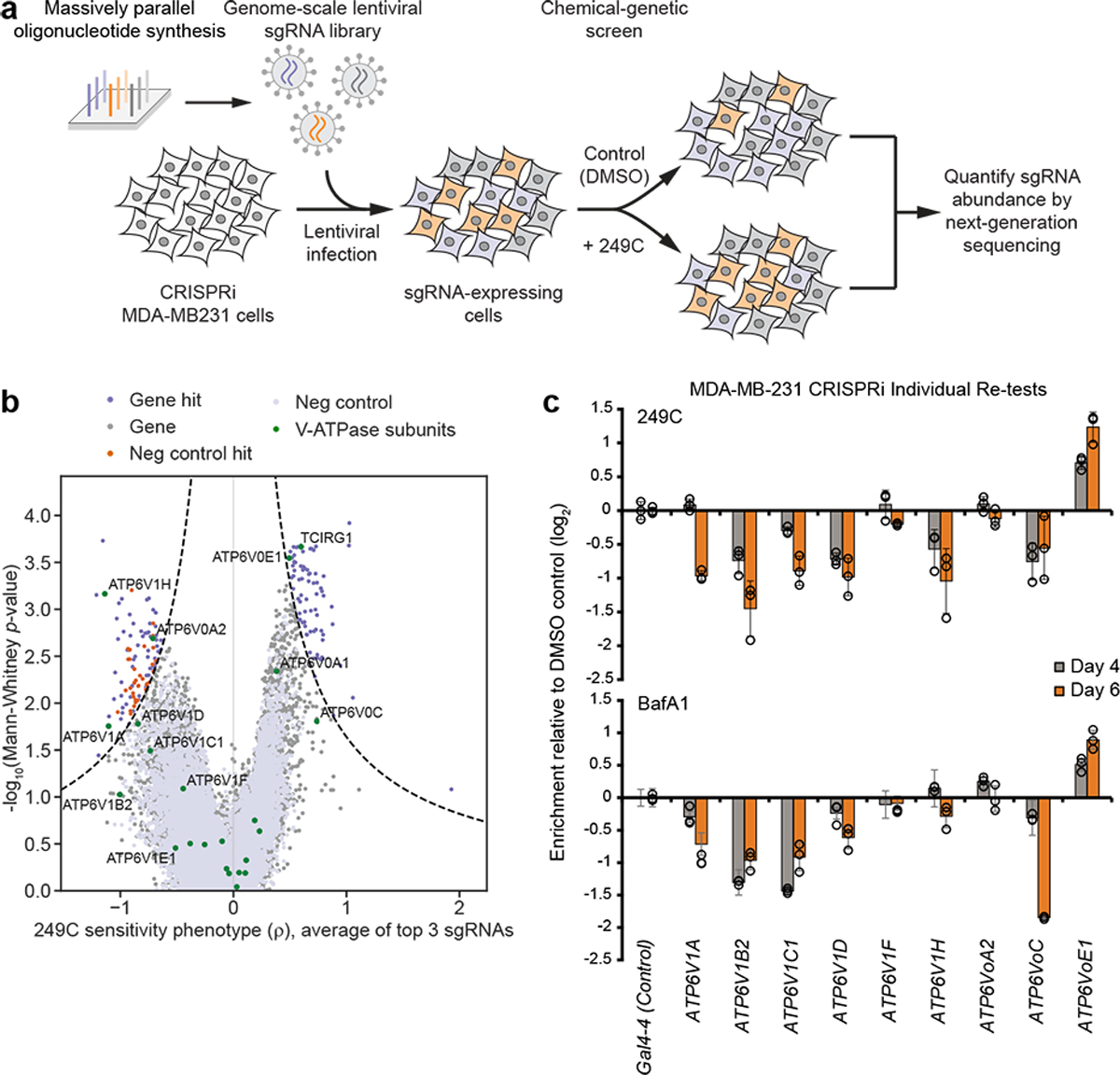
Genome-scale Chemical-genetic CRISPRi Screen Implicates V-ATPase as 249C’s Molecular Target. (**A**) Schematic illustration of the genome-wide CRISPRi chemical-genetic screen. (Created with BioRender) (**B**) Volcano plot of 249C sensitivity phenotype from genome-scale CRISPRi screen in MDA-MB-231. Phenotypes from sgRNAs targeting the same gene were collapsed into a single sensitivity phenotype for each gene using the average of the top three scoring sgRNAs and assigned a *p*-value using a two-sided Mann-Whitney U test of all sgRNAs targeting the same gene compared to the non-targeting controls. Negative control genes were randomly generated from the set of non-targeting sgRNAs. Dashed line represents discriminant score ≥7, calculated as phenotype *z*-score ∙ −log_10_(*p*-value), with the *z*-score defined from the standard deviation of the negative control genes. V-ATPase genes are shown in green. (**C**) Internally controlled individual re-tests for 249C and BafA1 sensitivity assays performed with sg-RNAs targeting (*ATP6V1 –A, B2, C1, D, F, H* and *ATP6V0 – A2, C, E1*) or a non-targeting control sg-RNA (*Gal4–4*) in MDA-MB-231 CRISPRi cells. Cells transduced with the sg-RNA expression constructs (marked with blue fluorescent protein [BFP]) were left untreated or treated with 249C/BafA1 4 days after transduction. Enrichment of sg-RNA-expressing cells was measured after treatment on the days indicated by flow cytometry as the enrichment of BFP (n = 3). Data presented: mean ± SD for replicate infections and treatments (n = 3). See also Fig. S4 and Supplementary Tables 5–6.

To further pinpoint the molecular target of 249C, we turned to CRISPR interference (CRISPRi)-mediated chemical-genetic screening, which has emerged as a robust strategy for small molecule target identification^23^. To allow us to work with a previously validated CRISPRi cell line, we evaluated a panel of available CRISPRi cell lines for sensitivity to 249C and found that breast adenocarcinoma MDA-MB-231 (*KRAS*G13D and *BRAF*G464V) were highly sensitive. Thus, we conducted these screens in MDA-MB-231 cells. Briefly, we infected MDA-MB-231 cells stably expressing the dCas9-KRAB CRISPRi effector^24^ with the genome-scale hCRISPRi-v2 single-guide RNA (sgRNA) library (targeting 18,905 genes)^25^, harvested a subpopulation at the outset of the experiment (t_0_), and then cultured the remaining cells without (DMSO) or with 249C treatment (Methods). Finally, we used next-generation sequencing to determine how each sgRNA impacted sensitivity to 249C (ρ) and growth in the absence of 249C (γ) (Fig. 3a)^26^. The resulting untreated growth phenotypes were well-correlated with those from other cell types, suggesting that our screen faithfully captured phenotypes resulting from gene knockdown (Fig.S4). Among the 147 genes for which knockdown strongly affected 249C sensitivity (Fig. 3b, Tables S5&6), we were intrigued to find 5 V-ATPase subunits (*ATP6V1H, ATP6V1A, ATP6V0A2, ATP6V0A3/TCIRG1*, and A*TP6V0E1*), because 249C essentially phenocopied the V-ATPase inhibitor BafA1 in our previous assays (Fig. 2c). Indeed, knockdown of several V-ATPase subunits sensitized cells to 249C; the regulatory H subunit ATP6V_1_H in particular was one of the most strongly sensitizing hits. By contrast, knockdown of the poorly characterized subunits *ATP6V0E1* and *ATP6V0A3* protected cells against 249C. Individual re-tests using internally controlled drug sensitivity assays further confirmed that knockdown of multiple V-ATPase subunits sensitized MDA-MB-231 cells to 249C and BafA1 (target: ATP6VoC)^27^ (Fig. 3c). Thus, genetic depletion of V-ATPase sensitizes cells to 249C, further suggesting that 249C’s cytotoxicity arises from inhibition of V-ATPase activity. Although we cannot rule out polypharmacology, the V-ATPase complex stood out based on multiple subunit hits.

### 249C binds and inhibits V-ATPase

**Figure 4.**
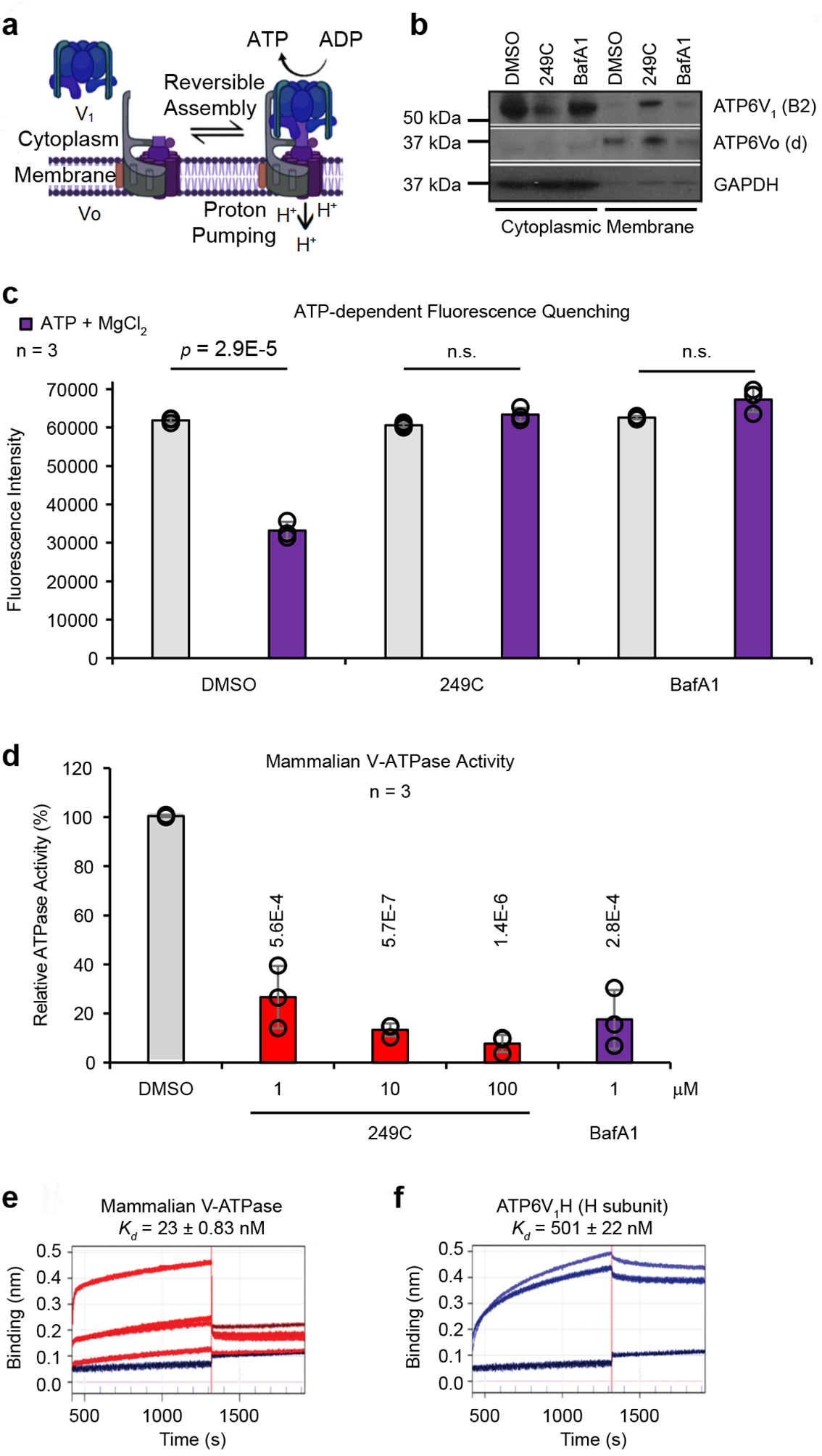
Validation of Functional Biochemical and Biophysical Effects of 249C on V-ATPase Activity. (**A**) Model of the reversible assembly of V-ATPase. Cytosolic V_1_ subunits are depicted in *blue* and membrane Vo subunits in *purple*. Upon V-ATPase assembly, ATP is hydrolyzed to ADP accompanied by proton (H^+^) pumping and luminal acidification (decrease in pH) (Created with BioRender). (**B**) HEK293T cells were treated with 249C or BafA1 and cell homogenates were prepared, separated into membrane and cytosolic fractions and analyzed by Western blotting using antibodies against subunit B2 as a measure of V_1_, subunit Vod as a loading control for the membrane fraction, and GAPDH as a loading control for the cytosolic fraction (see Methods). The amount of subunit B2 present in the membrane fraction indicates the amount of assembled V-ATPase. A representative Western blot is presented. (**C**) After 1 h of 249C or BafA1 treatment, HEK293T cells were allowed to take up FITC-Dextran by endocytosis, and the dye was chased to the lysosomal compartment (see Methods). Cells were mechanically lysed, and a fraction containing FITC-Dextran-loaded lysosomes was isolated by sedimentation centrifugation. Fluorescence was measured over time to assess pH-dependent quenching following addition of 1 mM magnesium-ATP (predetermined). ATP-dependent fluorescence quenching was not observed for 249C- or BafA1- treated samples. Representative of 5 individual experiments with n = 3 for each is presented (mean ± SD) (**D**) Mammalian V-ATPase activity measured in the absence and presence of 1 μM 249C and 1 μM BafA1. Data shown are mean ± SD; n = 3. Bio-layer inferometry (BLI) of (**E**) the entire mammalian V-ATPase complex (association protein concentrations: 30, 60, 120, 240 nM) and (**F**) the individual human H subunit (ATP6V1H) (association protein concentrations: 16,800 and 21,000 nM) against biotinylated-249C loaded onto streptavidin sensor tips. A reference sensor was subtracted from the signal to blank the system. The ForteBio Octet® Software on the BLI system was used to calculate *K*_*d*_ s. The V-ATPase complex: *K*_*d*_ = 23 ± 0.83 nM; H subunit: *K*_*d*_ = 501 ± 22 nM. Representative raw traces are presented from two independent experiments. See Fig.S5f for experimental schematic.

**Figure 5:**
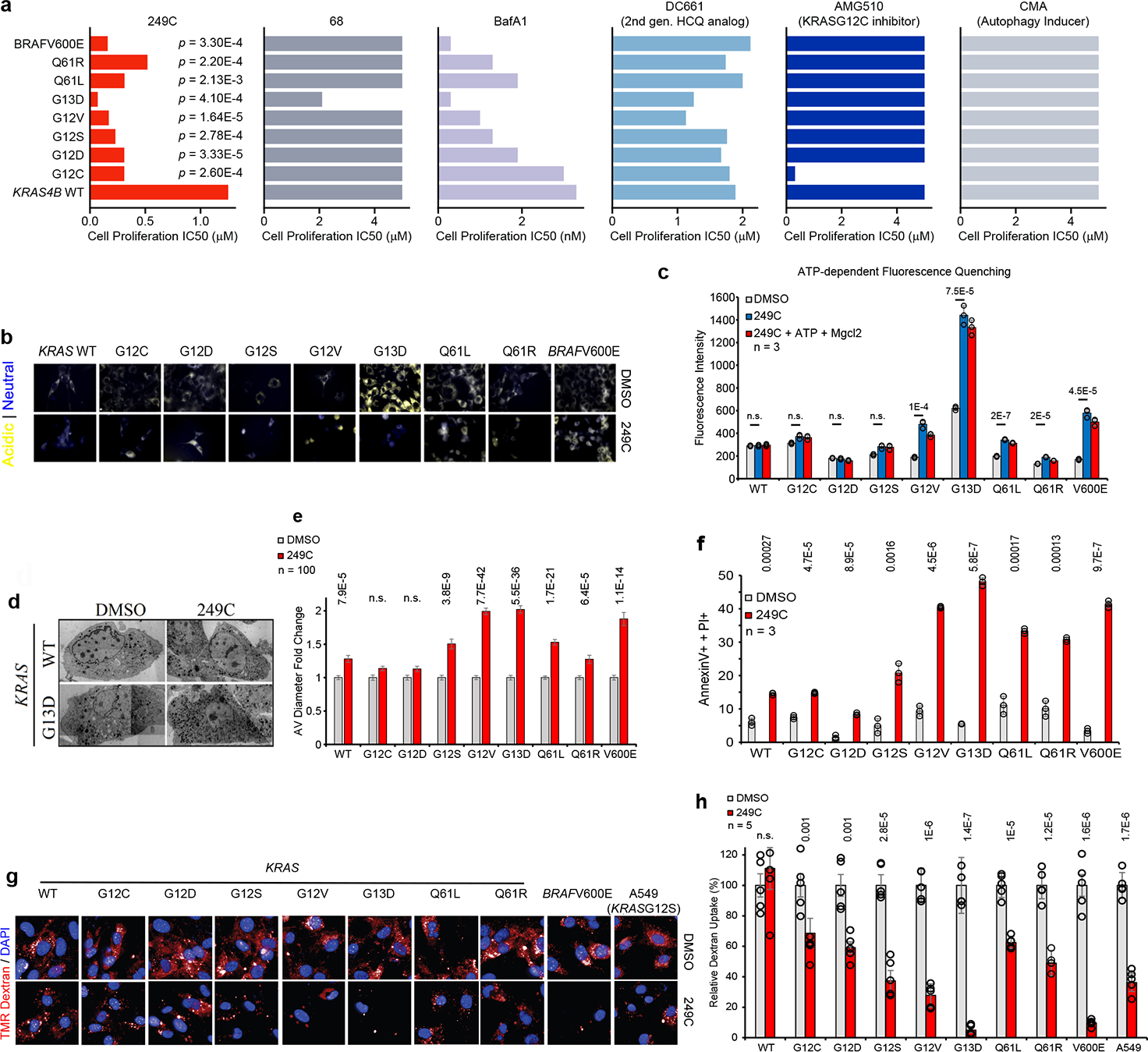
249C Differentially Inhibits Fibroblasts Bearing Mutations in *KRAS* and *BRAF* via Inhibition of lysosomal pH, V-ATPase activity, and Macropinocytosis. (**A**) Mouse embryonic fibroblasts (MEFs) bearing only single point mutations in human *KRAS/BRAF* treated with 249C. IC_50_ values: *KRAS*WT (1.25 μM), G13D (0.07 μM), G12V (0.15 μM), G12S (0.23 μM), G12D (0.3 μM), Q61L (0.31 μM), G12C (0.44 μM), Q61R (0.55 μM), and for BRAF*V600E* (0.11 μM). *p* values calculated relative to WT cells treated with 249C. Molecule **68** shown for comparison. Cell proliferation assays of stable cell lines treated with V-ATPase inhibitors 249C and BafA1, HCQ analog (DC661), *KRAS*G12C inhibitor (AMG510) and autophagy inducer (CMA). For each bar, n = 3. Representative of 3 biological replicates (mean ± SD). Two-sided *t*-test with no adjustments for multiple comparisons. Note: nM used for BafA1 (**B**) Basal levels of pH (yellow: acidic; blue: neutral | LysoSensor) in MEFs. Representative images from n >3. (see also Fig.S8). (**C**) After 2 h of 249C treatment (or DMSO), FITC-Dextran-loaded lysosomes were isolated from MEFs as in Fig. 3f. (see also Fig.S8 for DMSO+ATP+MgCl_2_ values). Representative of 2 individual experiments with n = 3 (mean ± SD). Two-sided *t*-test; n.s. not significant (**D**) Representative electron microscopy images for MEF *KRAS* WT and G13D showing multivesicular autophagic vesicles (AVs) after 250 nM 249C treatment for 20 h. Scale bars 5 μm. EM images were stitched together from multiple smaller frames with differences in contrast. (**E**) Mean fold change (±s.e.m.) of AV diameter (μM) in MEFs counted on electron microscopy images before and after treatment. *n*=100 cells per condition. Two-sided *t*-test. (**F**) Treatment with 100 nM 249C followed by quantification of double positive Annexin-V^+^/PI^+^ cells by flow cytometry at 48 hours relative to DMSO controls. Representative of 3 independent experiments. Two-tailed Student’s *t*-test. (**G**) Fluorescence micrographs showing tetramethylrhodamine (TMR)–dextran uptake after 249C treatment. (**H**) Quantification of TMR–dextran uptake. For each cell line, DMSO is set at 100%. The number of objects per nucleus was quantified using high-content imaging software (n = 3) data are mean ± stdv.; two-tailed Student’s *t*-test.

With multiple independent assays pointing toward V-ATPase as the target of 249C, we proceeded to validate the biochemical and biophysical effects of 249C on V-ATPase activity. V-ATPase activity depends upon reversible assembly of the V_1_ and Vo domains and is nutrient-dependent; increased V1-Vo assembly increases catalytic activity^15^. To test whether 249C alters localization of V-ATPase subunits, we evaluated the partitioning of V-ATPase subunits into the cytosolic [containing the V_1_ domain composed of eight subunits (A–H) responsible for ATP hydrolysis] and membrane fractions [membrane-embedded Vo domain comprised of six subunits (a, c, c′ (absent in mammals), c′′, d and e) responsible for proton translocation]^15^ of 249C-treated HEK293T cells. Treatment with 249C but not BafA1 led to an increase in membrane-associated subunit B2 (cytosolic V_1_ domain), possibly indicating increased V-ATPase assembly (Fig 4a–b). To independently corroborate the effect of 249C on V-ATPase and to probe whether proton pumping is impacted in the complexes on lysosomes, we measured lysosome acidification using a pH-dependent fluorescent probe, FITC-Dextran. Both 249C and BafA1 inhibited V-ATPase-dependent proton transport in mammalian cells, as measured by ATP-dependent fluorescence quenching of FITC-loaded lysosomes (Fig. 3c)^28^. Proton translocation through the Vo region is powered by ATP hydrolysis in the V_1_ region. In addition to blocking acidification, both 249C and BafA1 blocked ATP hydrolysis of intact mammalian V-ATPase purified from pig kidneys (but not yeast, Fig.S5) at concentrations as low as 1 μM, as measured by a standard biochemical V-ATPase activity assay (Fig. 3d). To directly measure binding of 249C to V-ATPase, we performed bio-layer inferometry (BLI) with both the purified intact mammalian V-ATPase complex and the recombinant ATP6V_1_H subunit using biotinylated-249C loaded onto streptavidin sensors (FigS.5e–f), which revealed binding with nanomolar affinities (*K*_*ds*_: complex = 23 nM; H sub = 501 nM) (Fig. 3e–f, Fig.S6). Altogether, these results establish that 249C directly binds to and inhibits mammalian V-ATPase.

### 249C preferentially kills mouse embryonic fibroblasts bearing specific *KRAS* mutations

The most frequent *KRAS* mutations across all human cancers occur at codons 12 or 13 with replacement of glycine to other amino acids: G12D (35%), G12V (24%), G13D (13%)^29^. To test the impact of 249C on cells carrying individual Ras/Raf mutations, we measured the potency of 249C against immortalized mouse embryonic fibroblasts (MEFs) genetically engineered to be “Ras-less” with subsequent stable transduction of human WT or mutant *KRAS* isoforms^30^ obtained from the NCI. The MEF system: 1) permits comparison of individual *KRAS* mutations in the absence of other oncogenic drivers found in cancer cells; 2) is routinely and widely used to examine the selectivity of *KRAS*-directed therapies^31–33^; and 3) is used to study autophagy^34^. Strikingly, 249C exhibited increased potency against cells expressing *KRAS/BRAF* mutants rather than the WT genes (Fig. 5a). Moreover, potency depended on the identity of the mutant [IC_50_ values: WT (1.25 μM), G13D (0.07 μM), G12V (0.15 μM), G12S (0.23 μM) G12D (0.3 μM), Q61L (0.31 μM) G12C (0.44 μM), Q61R (0.55 μM), *BRAF* V600E (0.11 μM)], suggesting that susceptibility to V-ATPase inhibition differs across *KRAS*/*BRAF* mutants (Fig. 5a and Fig.S1c| G13D p<0.05). Treatment with BafA1 showed similar profiles except for G12C, G12D, and Q61L, whereas the HCQ analog (DC661) and the autophagy inducer (CMA) showed substantial differences and the *KRAS*G12C inhibitor (AMG510) specifically killed cells expressing *KRAS*G12C. Of note, human and mouse ATP6V_1_H share 98.55% sequence identity; of the remaining, 5 amino acids are similar.

### 249C inhibits macropinocytosis in a *KRAS*-mutant-dependent manner

To investigate the mechanism of 249C’s mutant selectivity, we explored how 249C impacts four main phenotypes in MEFs expressing *KRAS/BRAF* mutants: 1) lysosomal pH; 2) dependence on V-ATPase activity; 3) apoptosis; and 4) levels of MP.

Cancer cells are reported to have lower lysosomal pHs than untransformed cells, and stably expressing oncogenic *RAS*G12V in untransformed cells is sufficient to decrease lysosomal pH^35^. To test this in our system, we stained MEFs bearing *KRAS* WT and mutants with a pH-sensitive dye and discovered that *KRAS*G13D expressing MEFs exhibited the most acidic lysosomes (basal levels) compared with WT or other mutants (yellow: acidic; blue: neutral | Fig. 5b). Thus the mere expression of *KRAS*G13D versus WT (or some of other mutants) renders the lysosomes in these cells more acidic. Since V-ATPases are responsible for acidifying lysosomes, these data indicate that *KRAS*G13D cells could bear more biochemically active V-ATPase. As expected, treatment with 249C or BafA1 increased the pH of lysosomes of *KRAS*G13D MEFs (Fig. 5b, Fig.S7). To next test if these differences in lysosomal pH were caused by differential dependence on V-ATPase activity, we measured lysosome acidification in MEFs bearing WT or *KRAS/BRAF* mutations using FITC-Dextran as in Fig. 3f. Untreated *KRAS*G13D MEFs accumulated the most FITC in their lysosomes, indicative of more active V-ATPase and thus more acidic lysosomes. As expected, 249C inhibited V-ATPase-dependent proton transport in most MEFs, as measured by ATP-dependent fluorescence quenching of FITC-loaded lysosomes (Fig. 5c; Fig.S8). Strikingly, 249C inhibited fluorescence quenching most strongly in *KRAS*G13D cells. V-ATPase inhibition and increased pH might subsequently halt the degradative process in lysosome-autophagic vesicle (AV) fusion. Indeed, when we analyzed these MEFs by transmission electron microscopy, we found considerably higher AV diameters in MEFs expressing *KRAS*G13D, *KRAS*G12V and *BRAF*V600E after 249C treatment compared with *KRAS* WT and other mutants, probably due to autophagic flux blockade (Fig. 5d–e). Next, as an orthogonal measure of cell death, when we assessed these MEFs by flow cytometry, we found that 249C induced apoptosis in MEFs expressing *KRAS*G13D (*p* = 0.0009), *KRAS*G12V (*p* = 0.002), and *BRAF*V600E (*p* = 0.001), but less so in MEFs expressing WT *KRAS* (Fig. 5f). Similarly, 249C significantly increased apoptosis in *KRAS* mutant cancer cells compared to WT (Fig.S8).

V-ATPase-dependent lysosome acidification is essential for autophagy and MP. We next tested if MP activity varies across different RAS/RAF mutant cells and whether such variations correlated with sensitivity to 249C. *KRAS*G13D (followed by G12V) cells had the highest levels of basal MP (absolute values) measured by uptake of fluorescently labelled dextran (Fig.S8a) suggestive of higher V-ATPase activity attributable to the identity of the *KRAS* mutant in these cells. Reciprocally, treatment with 249C caused the strongest inhibition of MP in MEFs bearing *KRAS*G13D (94%) and *BRAF*V600E mutations (90%), in agreement with the low IC_50_ of cells bearing these mutants [Fig. 5g–h | *KRAS*G12V (73%)]. Even though *KRAS*G13D is not associated with the highest proliferation rate, we speculate that of all available metabolic pathways, *KRAS*G13D cells derive a particularly high fraction of their energy from MP/autophagy and thus are particularly reliant on V-ATPase for survival.

### Sensitivity to 249C varies with MAPK, IGF1R, and EGFR signaling

We next asked whether differential dependence on other signal transduction pathways in different *KRAS* mutant cells contributes to 249C’s mutant specificity and whether such dependencies could be exploited for combination therapies. We first evaluated synergy between inhibitors of the MAPK signaling pathway and 249C, since concurrent inhibition of ERK1/2 and autophagy has been reported to synergistically suppress the growth of *KRAS*-mutant cells^36^. Using MEFs and cancer cell lines, we performed co-treatment with 249C and the following MAPK pathway inhibitors: AMG510 (*KRAS*G12C), Dabrafenib (*BRAF*V600), Trametinib (MEK1/2) and SCH772984 (ERK1/2) (Fig.S9a). As anticipated, *KRAS*G12C-mutant lines were sensitive to *KRAS*G12C-specific inhibition via AMG510, which was heightened when combined with 249C (Fig.S9b). Also, as expected, the *BRAF*V600E-mutant lines were sensitive to *BRAF*V600 inhibition via Dabrafenib, which was heightened when combined with 249C (Fig.S9c). Finally, Trametinib (MEKi) (Fig.S9d) and SCH772984 (ERKi) (Fig.S9e) alone modestly decreased proliferation in MEFs expressing *KRAS* mutants (left panel) and selected cancer cell lines with *KRAS* mutations (right panel). Co-treatment with 249C led to further decreased viability, especially at increased concentrations, with effects ranging from additive to mildly synergistic in different cell lines, suggesting that 249C-treated cells may transduce varying levels of signaling via the MAPK pathway. Thus, the addition of 249C could sensitize cells bearing less sensitive *KRAS* mutants to MAPK pathway inhibitors, expanding the potential therapeutic relevance of 249C. For example, Panc1 cells are not particularly sensitive to Trametinib individually, but respond well when combined with 249C (Fig.S9d).

We next also evaluated signaling through IGF-1R using an IGF-1R-specific inhibitor, AG-1024. MEFs expressing *KRAS*G12V or G13D were insensitive to AG-1024, likely due to a lack of signaling through this pathway (Fig.S10a). In comparison, MEFs expressing WT *KRAS* and other mutants (G12C, G12D, G12S, Q61L, Q61R, V600E) were sensitive to inhibition, indicating that they can readily signal through the insulin signaling pathway, which can promote cell proliferation (Fig.S10b). Thus, we speculate that upon V-ATPase inhibition, cells bearing WT *KRAS* and other mutants could signal through an alternative pathway for survival, but cells bearing *KRAS* G12V and G13D cannot rely on the insulin pathway.

Finally, we explored the effect of EGFR inhibition using Erlotinib. Consistent with previous reports^37–40^, we found that EGFRi was strongest for cells expressing *KRAS*G13D and WT in both MEFs and SW48 cells, indicating their dependence on EGFR signaling, whereas other mutants were not as responsive (FigS.10c–d). Since *KRAS*G13D has been suggested to maintain dependence on EGFR signaling compared to other *KRAS* alleles, we investigated the effects of 249C treatment on EGFR localization via immunofluorescence. We found that in 249C-treated *KRAS*G13D cells, EGFR localized predominantly in a punctate manner, likely in vesicles, but in cells expressing WT *KRAS* and other mutants the staining pattern was diffuse (FigS.10 red arrows).

WT *KRAS* and mutants other than G12V, G13D, *BRAF*V600E appear to have alternative signaling capabilities that allow these cells to survive in 249C’s presence. Vice versa, 249C appears to target a composite signaling “Achilles’s heel” for the sensitive mutants.

### 249C inhibits tumor growth in vivo with minimal toxicity

**Figure 6:**
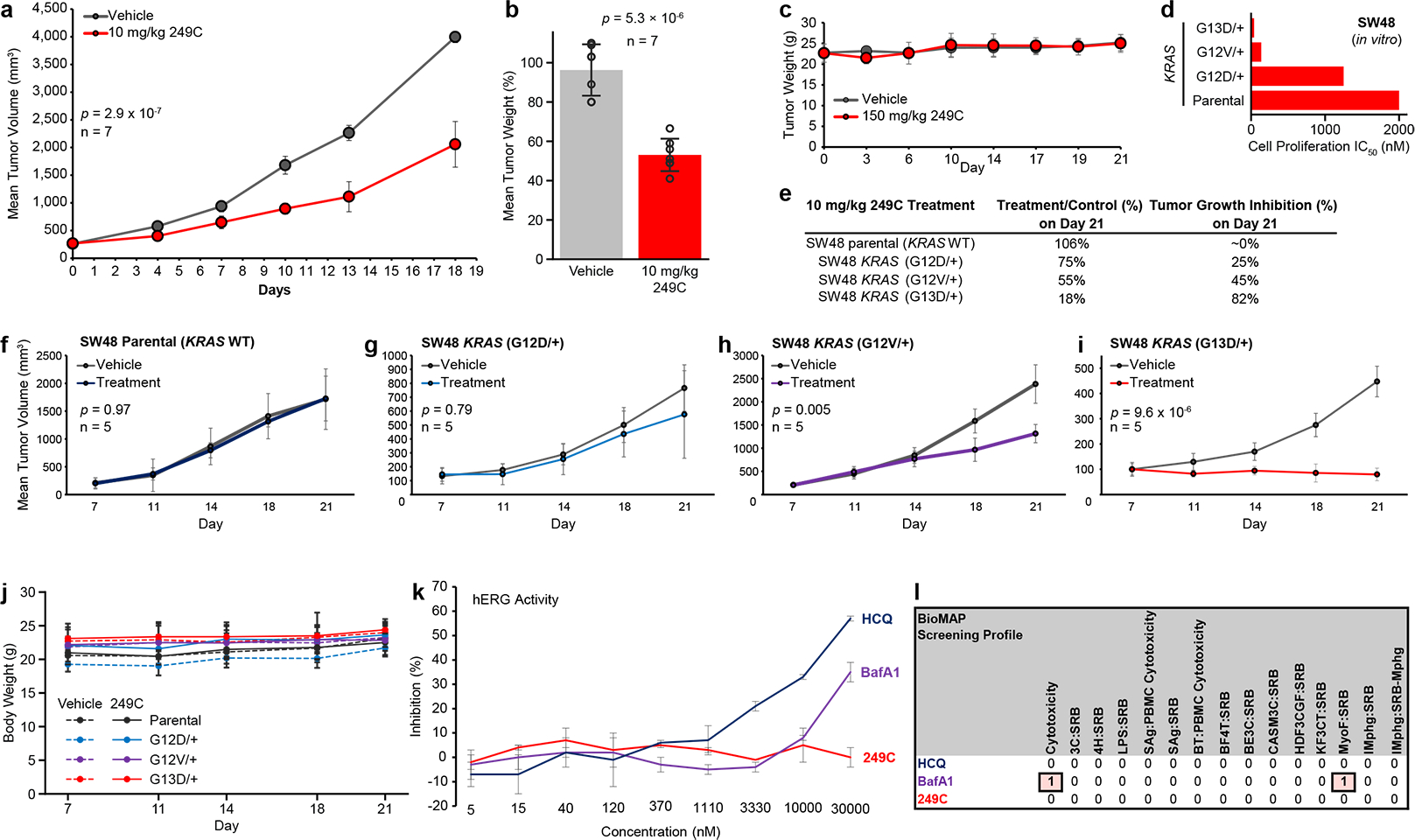
249C Treatment Inhibits *in vivo* Growth of Mutant *KRAS* in Mouse Xenograft Models and shows Pharmacodynamic Safety. (**A**) 5-week-old athymic mice were inoculated with 5 × 10^6^ NSCLC A549 (*KRAS*G12S) cells in the lower flank region to establish tumors and randomly assigned to treatment regimens of DMSO vehicle control or 249C (10 mg/kg × twice a day, i.p.). Changes in tumor volume of mice treated with the vehicle control or 249C (n = 7, *p* = 2.9 × 10^−7^) over the course of the study are shown. Two-sided *t*-test. (**B**) At the end point, tumor mass was determined on harvested tumors by weighing (n =7, *p* = 5.3 × 10^−6^). Two-sided *t*-test. (**C**) The safest highest dose of 249C measured is 150 mg/kg as no significant difference in body weight was observed after 21 days *in vivo*. (n = 7) (**D**) ***in vitro and*** (**E**) ***in vivo* activity of 249C in SW48 isogenic xenografts:** Antitumor activity of 249C on tumor volume in SW48, SW48 *KRAS*G12D/+, SW48 *KRAS*G12V/+ and SW48 *KRAS*G13D/+ xenografts presented as treatment/control (%) and tumor growth inhibition (TGI %) at Day 21. Tumor growth of SW48 colon tumor xenografts over time for vehicle control and 249C treated animals bearing isogenic SW48 (parental, *KRAS*G12D/+, *KRAS*G12V/+, *KRAS*G13D/+) cells in athymic mice. Changes in tumor volume for SW48 (*p* = 0.97) (**F**), SW48 *KRAS*G12D/+ (*p* = 0.79) (**G**), SW48 *KRAS*G12V/+ (*p* = 0.005) (**H**), and SW48 *KRAS*G13D/+ (*p* = 9.6 × 10^−6^) (**I**) after 14 days of treatment (10 mg/kg 249C or vehicle control, i.p., n = 5 for all arms). Two-sided *t*-test. (**J**) Body weight (g) of the mice bearing SW48 cells over 21 days. (**K**) Percent inhibition in fluorescence polarization assays of human *ether-à-go-go*-related gene (hERG) by HCQ (~60% at 30 μM), BafA1 (35% at 30 μM) and 249C (0% at 30 μM) (mean ± stdv; n=3) (see also Fig. S9). (**L**) *in vitro* BioMap® Safety and Toxicology screening profile of HCQ, BafA1 and 249C at 1000 nM across over 100-panel biomarker readouts. Data are mean ± stdv.; two-tailed Student’s *t*-test for all graphs presented.

Finally, to test whether 249C could inhibit autophagy and attenuate the growth of mutant *KRAS*-dependent cancers as a single agent *in vivo*, we evaluated 249C in a mouse xenograft model of lung cancer (NSCLC, A549). Briefly, we injected mutant *KRAS* A549 (G12S) cells into the flanks of athymic mice to establish xenografts, randomly assigned mice to vehicle control (n = 7) or 249C-recipient groups (n = 7, 10 mg/kg) with twice daily i.p. injections, and recorded tumor volume and body weight over time. During the course of the study, all mice survived but tumor volume was significantly lower in 249C-treated mice (Fig. 6a, *p* = 2.9 × 10^−7^). We subsequently sacrificed all mice and excised their tumors for further investigation. Tumor mass was lower in the 249C-treated group (Fig. 6b, *p* = 5.3 × 10^−6^), and tumors from mice treated with 249C showed upregulation of LC3-I/II, as assessed by immunoblotting, indicative of inhibition of autophagic flux (Fig.S11). Pharmacokinetic studies revealed that after 4 hours, a safe maximum concentration (C_max_) of 20 μM of 249C was detected in blood (or plasma) of mice, confirming metabolic stability (Fig.S11). Furthermore, 150 mg/kg of 249C was determined to be the maximum tolerated dose in mice and none of the following changed in either group: 1) body weight (Fig. 6c); 2) internal organ tissue weights; and 3) blood cell counts (Fig.S11) suggesting a lack of toxicity from treatment; yet even a dose as low as 10 mg/kg is sufficient for antitumor activity.

To explore the relationship between individual *KRAS* mutants and 249C sensitivity *in vivo*, we used a colon cancer (SW48) xenograft model with a set of colon cancer cell lines which differ in *KRAS* mutation status but are otherwise genetically matched. Stable derivatives of the SW48 cell line were derived by introducing a *KRAS*G12D, *KRAS*G12V or *KRAS*G13D point mutation via rrAV gene editing technology previously reported^41^. To compare *KRAS* mutants in a cancer-relevant system, four cell lines: (SW48, SW48 *KRAS*G12D/+, SW48 *KRAS*G12V/+ and SW48 *KRAS*G13D/+) were injected into athymic mice and 14 days of treatment (10 mg/kg 249C or vehicle control, i.p., n = 5 for all arms) was started when the average tumor volume reached approximately 100–200 mm^3^ following random segregation into groups. As before, *KRAS*G13D tumors were most sensitive to 249C both *in vitro* and *in vivo* (Fig. 6d–e). On day 21, 249C’s antitumor activity, presented as treatment/control, was 18% for SW48 *KRAS*G13D/+ (*p* = 9.6 × 10^−6^), 55% for SW48 *KRAS*G12V/+ (*p* = 0.005), 75% for SW48 *KRAS*G12D/+ (*p* = 0.79), and ~100% for parental SW48 (*p* = 0.97), and the corresponding tumor growth inhibition (TGI) was 82%, 45%, 25% and 0% respectively (Fig. 6e). Changes in tumor volume are presented for animals bearing isogenic SW48 cells: parental/*KRAS*WT (Fig. 6f), *KRAS*G12D/+ (Fig. 6g), *KRAS*G12V/+, (Fig. 6h), *KRAS*G13D/+ (Fig. 6i). Note that SW48 tumors with different *KRAS* variants show different basal growth rates, as reported previously^42^. Nonetheless, these data indicate that replacement of a single WT allele with a mono-allelic *KRAS*G13D/+ mutation is sufficient to significantly and drastically alter sensitivity to 249C and result in tumor regression in a xenograft model. Further, no significant changes in body weight of the mice were observed during the study (Fig. 6j).

To further evaluate the potential for secondary effects and adverse events in human cells, which is key to preventing Phase I failures, we assessed the effect of 249C on human hERG. Upon side-by-side experimental comparison with other autophagy modulators, we show that 249C is significantly less toxic than BafA1 and HCQ. Whereas the autophagy inhibitors CQ and HCQ are known to induce a high risk of cardiac electrocardiogram Long QT Syndrome (LQTS) through inhibition of hERG, 249C showed no risk at 30 μM (0% inhibition) (Fig. 6k), whereas HCQ showed ~60% inhibition, BafA1 displayed 35% inhibition, and DC661 showed 100% inhibition (Fig.S11) indicating less risk and a sizeable therapeutic window for 249C^18,43^. Finally, we used BioMAP® Phenotypic Safety and Toxicology profiling^44^ to screen for adverse effects. In human primary cells relevant to physiology, BafA1 showed toxicity in 2 categories but 249C was determined to have no adverse events across over 100 biomarker readouts, an endorsement of its candidacy to progress into the clinic (Fig. 6l). Collectively, these data indicate that 249C is efficacious in inhibiting tumor growth with minimal side effects (Fig.S12) in our investigations thus far, establishing *in vivo* proof-of-concept for targeting autophagy/MP/lysosomal acidification with 249C in mutant *KRAS* cancers. Interestingly, while profiling for off-target kinase hits (Fig.S12), we found that 249C bound to a tyrosine kinase called DDR1, which has been suggested as a therapeutic target for PDAC^45–46^. Very recently DDR1 was reported to get activated to compensate for loss of *KRAS* signaling^47^ implying that combination therapy of 249C with RAS pathway inhibition could results in synergistic activity as observed in Fig.S8.

## Discussion

In this study, we describe the discovery, target identification, and mechanism of action of 249C, a lead compound for treatment of *KRAS*G13D and G12V mutant cancers. 249C kills cancer cells by targeting a druggable hotspot within the V-ATPase subunit ATP6V_1_H and thereby inhibiting biochemical activity, lysosomal acidification, and macropinocytosis. Although 249C inhibits global cellular functions that depend on V-ATPase, we uncover that cells expressing *KRAS*G13D and *KRAS*G12V are particularly dependent on V-ATPase, creating a synthetic vulnerability exploited by 249C. With promising *in vivo* activity, low toxicity, and favorable pharmacokinetics, 249C is an ideal candidate for the development of patient-tailored drugs to treat cancers driven by *KRAS*G13D and *KRAS*G12V. In addition, the synergy of 249C with inhibitors of MAPK signaling in some *KRAS* mutant backgrounds provides an opportunity to explore new combination therapies.

Identification of the relevant mechanisms of action and cellular targets has been a critical barrier to exploiting small molecules with therapeutic potential from being developed into approved drugs. Here, three independent assays – chemo-proteomics, comparative profiling, and CRISPR-based chemical-genetic screens – pointed to V-ATPase as the molecular target of 249C, which we then confirmed using cell-based, biochemical and biophysical techniques. Compellingly, 249C directly bound the V-ATPase subunit ATP6V_1_H and inhibited proton pumping and ATP hydrolysis. ATP6V_1_H is part of the stator complex^48^ and is suggested to regulate bridging of rotor (Vo) and stator (V_1_) complexes and physically prevent ATP-driven rotation. 249C may alter V_1_-Vo bridging by binding to ATP6V_1_H. The physical coupling model proposed here is in alignment with previous reports of blocking V-ATPase using BafA1/ConA^49,50^.

A striking feature of 249C is the selectivity for *KRAS* mutants, which we established using 3 separate systems: 1) a panel of 53 established cancer cell lines (Fig. 1d & FigS.1c); 2) MEFs expressing specific *KRAS* alleles (Fig. 5a) and; 3) 4 × SW48 colon cancer cells/xenografts that differ in *KRAS* mutation status but are otherwise genetically matched (isogenic)[*in vitro* (Fig 6d–e) and *in vivo* (Fig 6f–i)]. Together, these 3 systems provide consistent and complementary evidence for the selectivity of 249C for *KRAS*G13D/G12V and highlight a fundamental vulnerability of G13D/G12V mutants that has not been reported in this context before.

This selectivity was not explained by differences in V-ATPase localization with different *KRAS* mutants (FigS.8), as might have been expected from previous reports^10^. Instead, this selectivity appears to arise from multiple converging factors. First, *KRAS*G13D and G12V cells have particularly high basal levels of MP and low lysosomal pH, suggesting that these cells derive a larger fraction of their energy from degradative processes such as autophagy and macropinocytosis and thus are particularly sensitive to V-ATPase inhibition. Second, cells expressing *KRAS* alleles other than *KRAS*G13D or G12V appear to derive proliferative signaling from additional pathways including the IGF-1R pathway, which may allow these cells to better withstand the presence of 249CFinally, *KRAS*G13D cells rely more strongly on EGFR signaling for growth, and 249C treatment disrupts EGFR localization, removing this pro-proliferative pathway.

Additional factors may contribute to 249C’s selectivity. For example, V-ATPase interacts with the Ragulator, which regulates mTORC1 activation and autophagy^51^. Thus, in addition to inhibiting autophagy and macropinocytosis, 249C may also disrupt compensatory mTORC1 signaling that normally occurs in these situations. Cells expressing 249C-insensitive mutants like *KRAS*G12D and G12C may also have alternate metabolic signaling pathways that promote survival, whereas cells expressing *KRAS*G13D, which derive most of their energy from lysosomal degradative processes, do not. In addition, differential changes in cellular pH from 249C across different *KRAS* mutants may affect downstream transcription factors differentially, which may further contribute to the inability of cells expressing *KRAS*G13D and G12V to engage other survival-promoting signal transduction pathways. It may be interesting to deconvolve how cells expressing *KRAS*G12V and *BRAF*V600E, which are sensitive to changes in pH due to 249C, differ in their signaling requirements from cells expressing *KRAS*G13D.

249C differs from known V-ATPase/autophagy inhibitors in both mechanism and pharmacology. Antifungal V-ATPase inhibitors, such as BafA1 and ConcanamycinA (ConA), target ATP6VoC, and other autophagy inhibitors, such as the antimalarials HCQ and CQ, act through a different target, PPT1. While both antifungals and antimalarials have potential therapeutic activity, their clinical utility is hampered by toxicity. BafA1 and ConA have been relegated as laboratory compounds, and HCQ and CQ show substantial off-target activity on hERG. By contrast, 249C shows minimal cytotoxicity compared to BafA1 and minimal inhibition of hERG while maintaining nanomolar potency. Further, BafA1 was first isolated in 1983 and to date no clinical trials have been launched. By contrast, 249C has already entered Phase I clinical trials.

Available evidence, such as differences in signaling in cells expressing different Ras mutants^36^,^52–54^, increasingly suggests that there may not be a single anti-Ras therapeutic approach for all RAS mutant cancers; instead, mutation-specific therapeutic strategies must be deployed for different RAS mutations. While the emergence of the covalent *KRAS*G12C inhibitor being developed by Amgen has garnered enthusiasm, there remains a pressing need for therapeutic strategies targeted at cancers driven by other *KRAS* mutations, especially given the dismal outcomes for patients with cancers driven by *KRAS* codon 13 mutations^55,56^. The G13D and G12V mutations account for 13% and 24% of *KRAS* mutations across all human cancers, respectively. Thus, 249C could become an effective therapeutic for ~37% of all *KRAS* malignancies. Beyond *KRAS*-driven cancers, autophagy dysregulation has been linked to a wide range of diseases including metabolic disorders, aging, and neurodegenerative diseases^57^, motivating investigation of 249C as a drug candidate for other treatment applications.

## METHODS

### Cell lines, Reagents and Proliferation Assays

Most cells were obtained from American Type Culture Collection (ATCC) and were cultured in ATCC-recommended media supplemented with 10% fetal bovine serum and 2% antibiotics (Penicillin-Streptomycin). MDA-MB-231 CRISPRi cells were a gift from Hani Goodarzi at UCSF (parental cells from ATCC). MEFs expressing human *KRAS/BRAF* were from the Frederick National Laboratory for Cancer Research, The National Cancer Institute. SW48 cells were obtained from Horizon Disc.

ATCC cell lines: H1944 (CRL-5907), A427 (HTB-53), A549 (CRM-CCL-185), SW1271 (CRL-2177), H292 (CRL-1848), H358 (CRL-5807), H1299 (CRL-5803), H460 (HTB-177), H1703 (CRL-5889), H2170 (CRL-5928), H2452 (CRL-5946), NCI H82 (HTB-175), Calu-3 (HTB-55), H838 (CRL-5844), H1975 (CRL-5908), H1650 (CRL-5883), H28 (CRL-5820), CFPAC-1 (CRL-1918), Capan-1 (HTB-79), Panc 02–13 (CRL-2554), ASPC1 (CRL-1682), Panc1 (CRL-1469), BxPC3 (CRL-1687), MDA-MB-231 (HTB-26), Sk-Br-3 (HTB-30), MCF7 (HTB-22), MDA-MB-436 (HTB-130), HCT116 (CRL-247EMT), Colo201 (CCL-224), N87 (CRL-5822), RKO (CRL-2577), SW48 (CCL-231), CaCO2 (HTB-37), SkMel5 (HTB-70), SKMEL30 (HTB-73), A375 (CRL-1619), A2058 (CRL-11147), DUI145 (HTB-81), PC3 (CRL-3471), U251 (HTB-17), U87 (HTB-14), HL-60 (CCL-240), HepG2 (HB-8065). LOX IMVI, MelJuso, BT474 and HCC1937 cells were a gift from the Oritz-Urda lab at UCSF originally sourced from ATCC or as gifts. ES2, OVCAR8, HEYA8, and HEY cells were a gift from the Smith-McCune lab at UCSF originally sourced from ATCC.

BafA1, Chloroquine, Hydroxychloroquine, and DC661 were from Medchemexpress LLC. Rapamycin was from Selleckchem and Torin was from Fisher. Erlotinib, AG-1024, and MAPK inhibitors were from Selleckchem. DNA transfection into cells was performed with TransIT®-LT1 Transfection Reagent (Mirus Bio) by following the manufacturer’s recommendations.

Logarithmically growing cells were plated in antibiotic-free medium supplemented with 2% fetal bovine serum at a density of 5,000 cells per well. The next day, cells were treated in triplicates with increasing doses of in-house small molecule inhibitor drugs and a DMSO vehicle control for 3 days and subsequently assessed for cell viability by measurement of ATP with CellTiter-Glo Luminescent Cell Viability Assay (Promega). Signal intensity was read on a Glomax™ 96 Microplate Luminometer (Promega) with GLOMAX software (version 1.9.3) and percent cell survival was calculated based on the reading of vehicle control cells set as 100% using Excel (2012) and GraphPad Prism (version 6)Each compound was tested a minimum of 2 times with n = 3. All cell proliferation assays (cancer cell lines or MEFs) were done in a similar way.

### Chemical Synthesis of 249C

249C and other small molecules (~300) were synthesized at UCSF and are covered by patent number PCT/US2017/039806 filed with the USPTO. Briefly, small molecules to represent diversity in the compound library were synthesized by the synthesis method described in Fig.S1. The synthetic route is designed to investigate functional group tolerability around the dihydro-pyrazole pharmacophore; simultaneous optimization was carried out for potency and physico-chemical properties. Analytical evaluation of compounds synthesized is presented in Table S3.

### Structure-Activity Relationship (SAR) Investigation

Compounds synthesized in this series are potent in multiple cancer cell lines. Most calculated properties meet drug-likeness criteria. Initial compounds are hydrophobic in nature, with relatively high logP and have low polar surface are. The lipophilicity of the compounds can be reduced via the introduction of polar side chains resulting in analogs with more favorable physico-chemical properties, i.e. drug likeness.

### Mutation Analysis in CCLE Cell Lines

The Cancer Cell Line Encyclopedia (CCLE, http://www.broadinstitute.org/ccle) was used to download somatic mutation data for over 1000 cancer cell lines [(CCLE_DepMap_18q3_maf_20180718.txt) from CCLE at https://depmap.org/portal/download/ (2018)]^61^. We filtered the data to retain only mutations (single nucleotide variants, SNVs, and insertion deletions, indels) detected in 53 cell lines that were included in our MTS assays (Fig. 1). Non-coding variants, silent mutations and in-frame insertions were removed, as these were not considered functional. We retained in-frame deletions as these may remove essential binding sites. The Combined Annotation Dependent Database (https://cadd.gs.washington.edu/) (version 1.6) was used to assess the predicted functional importance of SNVs, and we retained only variants with CADD Phred scores ≥15^62^. Including these predicted damaging SNVs as well as indels (minus in-frame insertions), we determined the number of times each gene was mutated across the 53 cell lines. Genes with multiple mutations in the same cell line were only counted once. Multiple Wilcoxon rank sum tests were carried out, using the “lapply” looping function in the R statistical environment, to test for association between presence/absence of mutations in each gene and IC_50_ values in each cell line. A Wilcoxon rank sum test was also carried out for Ras/Raf mutations, which included combined mutation data for genes *KRAS*, *NRAS*, *HRAS*, *NF1* and *BRAF*.

### Mass Spectrometric Whole Proteome Analysis

After treatment with small molecules, A549 cells were trypsinized, lysed in 8 M Urea, 1% SDS, 50 mM Tris pH 8.5, protease (Complete) and phosphatase (PhosStop) inhibitors, and samples were sent to The Thermo Fisher Center for Multiplexed Proteomics at Harvard to be processed as described^63^. Briefly, extracts were purified by TCA precipitation, followed by labeling with TMT reagents (Thermo Fischer), and subsequently desalted by StageTips before liquid chromatograph–tandem mass spectrometry (LC–MS/MS) analysis. Data were collected using an Orbitrap Fusion Lumos mass spectrometer (Thermo Fisher Scientific, San Jose, CA) coupled with a Proxeon EASY-nLC 1200 LC pump (Thermo Fisher Scientific). For LC- MS/MS analysis, an MS^3^-based TMT method was used as previously described^64^. For mass spectrometry data processing and spectra assignment, an MS2 spectra assignment false discovery rate (FDR) of less than 1% was implemented by applying the target-decoy database search strategy. To quantify TMT reporter ion intensity, a 0.003 *m*/*z* window centered on the theoretical *m*/*z* value of each reporter ion was monitored for ions, and the maximum intensity of the signal to the theoretical *m*/*z* value was recorded. Reporter ion intensities were normalized by multiplication with the ion accumulation time for each MS2 or MS3 spectrum and adjusted based on the overlap of isotopic envelopes of all reporter ions. After the reporter ion signal was extracted, the isotopic impurities of the TMT reagent were corrected using the values specified by the manufacturer. Total signal-to-noise values for all peptides were summed for each TMT channel and all values were adjusted to account for variance and a total minimum signal-to-noise value of 200 was implemented.

### MsigDB

GSEA software (version 4.2.3) and Molecular Signature Database (MSigDB version 7.4) from http://www.broad.mit.edu/gsea was used to query the top 8 proteins from the Whole Proteome Analysis for GO gene sets in NCI-60 cell lines^65,66^.

### BioMAP and Benchmarking

BioMAP assays comprising 12 human primary cell-based systems (co-cultured cells simulating 12 different disease contexts) were treated in the presence or absence of test agents (known drugs and our 249C compound) as described^67^. All cells were from a pool of multiple donors (n = 2 – 6) and collected in accordance with appropriate regulatory protocols. Direct ELISA was used to measure selected biomarker level readouts (adhesion receptors, cytokines, enzymes etc.) and activity profiles (normalized data sets) were generated for each test agent. The resulting profiles from biomarker readouts were compared or ‘benchmarked’ to a known 4000-molecule database by statistical methods to identify similarities and mechanistic insights described previously^68^ to provide Pearson and Z scores.

### Immunoblotting

Protein extracts were prepared from drug-treated cells by lysis on ice for 20 minutes in M-PER Mammalian Protein Extraction Reagent (ThermoScientific) supplemented with protease and phosphatase inhibitors (Roche). Lysates were centrifuged at 16,000 RPM for 20 minutes at 4°C and the supernatants were assessed for concentration via a Bradford assay. 25–50 μg of protein extracts were used for gel electrophoresis followed by immunoblotting onto PVDF membranes. Blots were probed with the following primary antibodies: GAPDH (ThermoFisher, 1:20,000), RAS (1:1000), SQSTM1 (1:1000) and LC3 (1: 1000) (3 × Cell Signaling).

### Electron Microscopy

A549 cells attached to ACLAR film^69^ were treated for 24 hours with 249C (250 nM) or BafA1 (10 nM) or DMSO as a control. The cells on film were then fixed by immersion in 2% glutaradehyde in 0.08 M Na-cacodylate buffer, pH 7.3 containing 2 mM CaCl_2_ that had been prewarmed to 37 ° C for 1 hour with gentle agitation during which the fixative cooled to room temperature. The samples were then rinsed with 0.1 M Na-cacodylate buffer at room temp., post-fixed with 1% OsO_4_ containing 1.5% potassium ferrocyanide in 0.1 M Na-cacodylate buffer for 45 min on ice, rinsed with water, *en bloc* stained with 3.5% uranyl acetate in water for 1 hour at room temp., dehydrated in ethanol followed by propylene oxide, and embedded in Eponate 12 resin (Ted Pell, Inc, Redding, CA). Thin sections were cut with a Leica UCT ultramicrotome using a Diatome diamond knife and picked up on Pioloform films on slot grids. The grids were then post-stained with 1% uranyl acetate followed by Sato’s lead^70^. Sections were imaged with an FEI T12 TEM equipped with a Gatan U895 4k × 4k camera at 120 kV [software: DigitalMicrograph (version 3.4.3) and SerialEM (version 3.8.6)]. Quantification was performed with custom Python image analysis software. MEFs were processes in a similar way.

### Lysosome Staining

Cells were treated with the respective molecules in 2-well chambered coverglass slides at a final concentration of 1 μM for 18 hours followed by addition of lysosome-specific dyes. LysoTracker™ Red DND-99 and pH sensitive-LysoSensor™ Yellow/Blue DND-160 purchased from ThermoFisher were added to the cells for 1 hour at 37° C along with Hoescht to stain DNA according to the manufacturer’s recommendations. Live cell images were captured at 20X with a Zeiss spinning disc confocal & TIRF Fura microscope at UCSF’s Laboratory for Cell Analysis.

### CRISPR Screen

Genome-scale screens using MDA-MB-231 CRISPRi cells (a gift from Hani Goodarzi at UCSF) were performed in a similar manner as previously described^71,72^. For genome-wide knockdown, CRISPRi-v2 sgRNA libraries^72^ [targeting 18,905 genes and marked with Blue Fluorescent Protein (BFP)] were transduced into MDA-MB-231 CRISPRi cells at an MOI < 1 (percentage of transduced cells 2 days after transduction: ~30%). Cells were maintained in DMEM in 20 × T-182.5 flasks for the course of the screen. After transduction, the cells were selected with puromycin for 2 days, at which point transduced cells accounted for 90% of the population assessed as the fraction of BFP-positive cells by flow cytometry. Following 1 day of recovery without puromycin, samples at time-point t_0_ with a minimum established coverage of >1,000 cells/sgRNA were harvested and the remaining cells were split into two populations for untreated growth (DMSO control) and 249C-treated growth. The cells were maintained in T-182.5 flasks at an average coverage of greater than 1000 cells per sgRNA for the duration of the screen. For 249C treatment, 525 nM 249C was added to the cells at Day 0 and Day 5 and removed the following day. Cells were harvested on days 19 (DMSO) and 21 (249C) (6.1 doubling differences between treated and untreated populations). Genomic DNA was isolated and the sgRNA-encoding region was amplified and processed for next generation sequencing on an Illumina HiSeq 4000 (HiSeq Software Suite v3.4.0) as described previously^73^. Sequencing reads were aligned to the CRISPRi library sequences, counted, and quantified using the Python-based ScreenProcessing pipeline available at https://github.com/mhorlbeck/ScreenProcessing^72^. Generation of negative control genes and calculation of phenotypes and Mann-Whitney p-values was performed as described previously^71^. Sensitivity phenotypes (ρ) were calculated by calculating the log_2_ change in enrichment of an sgRNA in the treated and untreated samples, subtracting the equivalent median value for all non-targeting sgRNAs, and dividing by the number of population doubling differences between the treated and untreated populations^71,73,74^. Similarly, untreated growth phenotypes (γ) were calculated from the untreated and t_0_ samples, dividing by the total number of doublings of the untreated population. Phenotypes from sgRNAs targeting the same gene were collapsed into a single sensitivity phenotype for each gene using the average of the top three scoring sgRNAs (by absolute value) and assigned a p-value using the Mann-Whitney test of all sgRNAs targeting the same gene compared to the non-targeting controls. For genes with multiple independent transcription start sites (TSSs) targeted by the sgRNA libraries, phenotypes and p-values were calculated independently for each TSS and then collapsed to a single score by selecting the TSS with the lowest Mann-Whitney p-value. Read counts and phenotypes for individual sgRNAs are available in Supplementary Table 5. Gene-level phenotypes are available in Supplementary Table 6. All additional analyses were performed using python3.7 using a combination of Numpy (v1.15.4), Pandas (v0.25.3), and Scipy (v1.4.1).

### Individual CRISPR Re-tests

Oligonucleotides used to construct plasmids expressing sgRNA targeting the selected genes for individual re-tests were acquired from IDT and cloned into the pCRISPRia-v2 vector (Addgene #84832) backbone followed by DNA transformation, extraction, and sequencing to verify accuracy and packaging of lentivirus. After 5 days, transduced CRISPRi cells were left untreated (DMSO) or treated with 525 nM of 249C or 30 nM BafA1 for 24 h and assessed for blue fluorescent protein (BFP) using an LSR II flow cytometer at UCSF’s Flow Cytometry Core facility. Experiments were performed in triplicates. To validate screening results, we measured the effects of knocking down all 9 subunits on 249C sensitivity in individual re-tests. We infected MDA-MB-231 CRISPRi cells with constructs expressing sgRNAs targeting V-ATPase subunits (*ATP6V1 – A, B2, C1, D, H, F* and *ATP6V0 – A2, C, E1*) or a non-targeting control sgRNA (*Gal4–4*) and used flow cytometry to monitor how the fraction of sgRNA-expressing cells changed after treatment with 249C. MDA-MB-231 CRISPRi cells expressing ATP6V_1_H subunit targeting sgRNA were depleted the most compared to others (Fig. 3c), thus reproducibly indicating that *ATP6V1H* levels affect 249C sensitivity. Thus, 249C’s pattern of drug-gene interactions closely resembles those of V-ATPase-inhibiting agents, particularly that of BafA1, which binds to the ATP6VoC subunit^27^.

### Subcellular Fractionation

Cytoplasmic and membrane extracts from A549 cells left untreated (DMSO) or treated with 249C (20 μM) or BafA1 (1.5 μM) for 2 hours were prepared using a Subcellular Protein Fractionation Kit by following the manufacturer’s recommendations (Fisher). Fractions were subjected to Western blotting as described above and blotted for ATP6V1B2 (a gift from Dennis Brown at Harvard University, 1:1000) and ATP6VoD (Abcam, 1:1000) antibodies.

### Protein Purification

Mammalian V-ATPase complex: the entire V-ATPase complex (800 kDa) was purified as described previously from porcine kidneys^83^ using the detergent glycol-diosgenin.

ATP6V_1_H (H-subunit): Addgene Plasmid #14658 was expressed in Rosetta2(DE3)pLysS cells and induced with IPTG. Harvested lysates were loaded onto a GSTrap column and bound protein eluted with reduced glutathione. Thrombin was added to cleave the GST tag followed by loading onto a BioRad SEC650 size-exclusion column (see Fig.S5).

### Bio-Layer Interferometry Studies for Binding Affinity Determination

Biotinylated-249C was diluted in PBS + 0.05% Tween + 0.2% BSA, pH 7.4 and loaded onto Octet Streptavidin (SA) Biosensors (ForteBio) by following the manufacturer’s recommendations on an Octet RED384 machine (ForteBio, PALL Octet System). Biotin-249C immobilization was checked via Octet® Software prior to introduction of the entire V-ATPase complex and the individual H subunit protein diluted in blocking buffer (1X PBS + 0.05% Tween 20 + 0.2 % BSA + 10 μM Biotin) to eliminate non-specific binding. A reference sensor was subtracted from the signal to blank the system. The ForteBio Octet® Software (version12) on the BLI system was used to calculate the *K*_*d*_ values. Graphical output from the Octet® Software of representative data from two independent experiments is presented.

### Proton Pumping Assay in Mammalian Cells

HEK 293T cells were either left untreated (DMSO control) or treated with 10.5 μM 249C or 0.5 μM BafA1 for 1 hour at 37 °C. This assay was performed as described previously^75^.

4 × 10^6^ HEK 293T cells were plated in 10-cm plates. The next day, the medium was replaced with medium containing 2.2 mg/mL FITC-Dextran (Millipore Sigma FD40), to allow uptake of the dye by endocytosis. The following day, FITC-Dextran-containing medium was replaced with regular unlabeled medium that permits all dye to progress to the lysosomal compartment^76^. Cells were either left untreated (DMSO control) or treated with 15 μM 249C or 0.5 μM BafA1 for 1 hour at 37 °C. After treatment, cells were placed on ice, rinsed with ice-cold PBS and collected by scraping into fractionation buffer (125 mM KCl, 1 mM EDTA, 50 mM sucrose, 20 mM HEPES, 1 mM PMSF, and Halt protease inhibitors). Cells were collected by centrifugation at 1200 × g for 5 min, resuspended in fractionation buffer and lysed by passing through a 27-gauge needle 10 times. Cell lysates were cleared of nuclei and intact cells by centrifugation at 2000 × g for 10 min and the resulting supernatant was then centrifuged again for 15 min at 16,100 × g to sediment the FITC-Dextran-containing lysosomes^77,78^. The resulting pellets were resuspended in 100 μl of fractionation buffer and subjected to a Bradford assay to assess protein concentration^79^. To measure molecule-dependent proton pumping, 20 μg of protein was added to fractionation buffer pre-warmed to 37 °C. Sample fluorescence was excited at 490 nm, and emission fluorescence at 520 nm was measured continuously in BioTek plate reader (Synergy) using Gen5 software (version 3.05). After initial fluorescence stabilization, 1 mM ATP and 2 mM MgCl_2_ were added to initiate ATPase activity and proton pumping into the lysosomes, which causes quenching of FITC in the lysosomal lumen. The V-ATPase dependence of quenching was verified by adding 249C and BafA1, which inhibit fluorescence quenching. Representative of 5 individual experiments with n = 2 for each is presented. MEFs were processes in a similar way.

### Proton Pumping Assay in Yeast

Spheroplasts from wild-type yeast (a gift from the Walter lab at UCSF) were left untreated (DMSO control) or treated with 29 μM 249C, or 1 μM BafA1 for 2.5 hours with shaking at 30 °C and vacuolar membrane vesicles were isolated as previously described^80^. Proton transport was measured using ATP-dependent quenching of 9-amino-6-chloro-2-methoxy-acridine (Acridine Orange, ThermoFisher) fluorescence quenching for isolated vacuoles as previously described^81^

### Biochemical V-ATPase Assay

V-ATPase was purified as described previously from yeast^82^ using the detergent dodecylmaltoside and porcine kidney^83^ using the detergent glycol-diosgenin. ATPase assays with purified V-ATPase were performed as described previously. 249C (mammalian: 0.1, 1, 10, 100 μM and yeast: 5, 10 and 100 μM) or BafA1 (1 μM) were added to the reaction and compared with a negative control to which only DMSO was added. Enzyme-coupled ATPase biochemical activity assays were performed in a 96-well plate as described previously^84^.

### 3D Molecular Modeling, Binding Pocket Detection and Docking

A preliminary sequence alignment between the yeast (uniprot P41807) and human (uniprot Q9UI12) sequences, which share 27% homology/45% similarity, was generated with EMBOSS Stretcher on EMBL-EBI webserver. The alignment was then manually adjusted to avoid alignment gaps within secondary structural elements of the human protein, as predicted by the DISSPRED webserver (http://comp.chem.nottingham.ac.uk/disspred/). Next the human V-type proton ATPase, encoded by *ATP6V1H* was modeled based on the yeast homologue structure available in the PDB database (PDB ID #1HO8) using the MODELLER^85^ software version 9.19. The best modeled based on the DOPE^86^ score was used for subsequent virtual screening.

The Small Molecule Drug Discovery Suite 2019–1^87^ (Schrödinger, LLC) was used for binding pocket detection and virtual screening. The protein was prepared in a ready-to-dock-format with the *Protein Preparation Wizard* workflow. The hydroxyl group orientations and protonation states were assigned at pH 7 using *PROPKA* in *Epik v4.7*. The *SiteMap* algorithm was used to screen for potential binding pockets within the entire surface of the protein pocket, using standard parameters. Small molecules were drawn with *Maestro* v11.9, and their protonation states and tautomers assigned at pH 7.0 ± 2 in *Epik* v4.7. Conformers were assigned using *LigPrep* v4.9 with the OPLS3e force field. Next, *Glide* v.8.2 was used to generate a grid box for docking the small molecules into each detected binding site. Prior to docking, a 37 × 37 × 37 Å^87^ docking grid was erected. The Glide XP^88^ scoring function was employed, and the strain energy was included in the final docking score, a maximum of 10 docking poses was set for each ligand. Planarity of aromatic groups was enforced. Final binding poses were picked based on a combination of visual inspection, chemical intuition, and score.

### Macropinosome Visualization and Quantification

Macropinosome visualization was essentially done as previously described^89^. Cells were plated in black, clear bottom CellCarrier-96 Ultra Microplates (PerkinElmer 6055302) and serum-starved for 24 h. The next day, they were treated with 2 μM 249C (1255) for 18 h followed by addition of 70 kDa TMR-Dextran (Invitrogen D1818) and Hoescht for 2 hours at 37 °C prior to 3 × cold PBS washes and fixation in 4% paraformaldehyde for 30 min. Following 3 × cold PBS washes, mounting medium (Vector Laboratories Inc.) was added and images were captured using an Operetta CLS™ fluorescent microscope for High-Content Analysis (PerkinElmer). Image analysis and quantification was performed with a spot segmentation algorithm using the Perkin Elmer Harmony® PhenoLOGIC™ 3.2 software. Briefly, the images were segmented and individual nuclei (DAPI stained) and cell bodies (by digital phase contrast imaging) were identified. The TMR signal was used for the spot segmentation analysis block. Number of spots, area, and TMR fluorescence intensity were calculated for each cell region, and displayed as mean values per cell. Data are presented as spots - number of objects per nucleus. For each cell line, 5 DMSO control and 5 249C-treated wells were imaged and a minimum of 3 images were captured per well. Data representing three independent experiments are presented.

### Immunofluorescence Assays

Cells were seeded onto glass coverslips and subsequently serum-starved for 24 h. After serum starvation, cells were treated with 500 nM 249C for ~15 hours and fixed with 4% formaldehyde for 30 min at room temperature. The following sequential steps took place at room temperature: cells were washed twice with PBS, permeabilized (0.1% Tween in PBS) for 10 min, and blocked (5% bovine serum in PBS) for 30 min. The following primary antibodies were used: ATP6V1A (Abnova, H00000523-M02 1:250 dilution), ATP6V1B2 (ThermoFisher, PA552518, 1:50 dilution), ATP6V1H (PA552518, Fisher, 1:50 dilution), EGFR (4267P, Cell Signaling, 1:100 dilution). Corresponding alexafluor-488 secondary antibodies were used at 1:500 dilution (ThermoFisher, mouse: A11001, rabbit: A11008). Cells were mounted onto slides using Mounting Media (Vector Laboratories Inc.) containing DAPI. A minimum of 3 images per well were captured using a spinning disk confocal microscope (Zeiss with ZEN blue software (2012)). Data representing two independent experiments are presented.

### Plasma Membrane Fractionation

The plasma membrane protein extraction kit (BioVision, K268–50) was used to separate the plasma membrane fraction from other cellular membranes according to the manufacturer’s recommended protocol. Data representing two independent experiments are presented.

### Apoptosis Assays

Cells were treated with 100 nM 249C for 48 hours and following two washes with ice-cold PBS, they were stained using a FITC Annexin V/PI Apoptosis Detection kit (BD Pharmingen) by following the manufacturer’s recommendations. Samples were analyzed by flow cytometry on a BD™ LSR II flow cytometer with FACSDiva software (version 8.0). Gating Strategy: Preliminary FSC/SSC gates were applied followed by removal of doublets (FSC-H vs FSC-A and also SSC-H vs SSC-A). Next single color controls (FITC only or PI only) and double positive controls (FITC+ & PI+) were run to set gates for the double positive population. Finally, untreated and 249C-treated samples were run keeping these gates constant for all.

### Animal Studies, Plasma Concentration, Body Weight

5 × 10^6^ A549 cells were subcutaneously injected into the lower left flank of 6–8-week old athymic nude female mice (Charles River strain # 088, homozygous NU/NU). To assess establishment of tumors, mice were examined 5 days post-inoculation and were randomly segregated into two treatment groups [vehicle control, 10 mg/kg 249C IP, n = 7 per group] and were treated twice daily with the drug in NPM or vehicle control (5% weight aqueous solution of carboxymethyl cellulose). Tumor volumes were calculated using the formula (L × W × H)/2 with recorded caliper measurements. Survival curves and statistical analyses were performed using Excel. Animals were maintained on a standard chow diet; 12:12 dark/light cycle and in group housing in hepa-filtered cages. All animal procedures were performed under IACUC approved protocols and guidelines. Animals from both groups were weighed every 3 days. Plasma concentration was measured from blood collected using standard and established protocols.

For cell line xenografts, 6 × 10^6^ SW48 cells were subcutaneously injected into the flanks of 6–8-week old athymic nude female mice (Taconic model # NCRNU-F, CrTac:NCR-Foxn1 sp/sp) and tumor growth was monitored with caliper measurement. Once the mean tumor reached a certain volume (between 100–200 mm^3^), animals were randomized into different treatment groups of 5 animals per group. 249C for *in vivo* use was formulated in 0.5% Methylcellulose and dosed i.p. (10 mg/kg, for 2 weeks). Antitumor activity was determined by calculating the treatment over control (T/C) tumor volume ratio at the end of the study. Either at the end of each study, of if any animal reached a humane endpoint, animals were euthanized with CO_2_ followed by cervical dislocation or another secondary method of euthanasia approved by the IACUC protocol. Mouse manipulations were performed in accordance with UCSF’s IACUC protocol AN179973.Mice were housed in the UCSF Animal Care Facility LARC at the Helen Diller Family Cancer Center at UCSF Mission Bay. Mice were housed in a specific-pathogen free individual suite. They are housed up to 5 per cage in ventilator cages, with *ad libitum* food and water on a 12-hour light cycle and controlled temperature and humidity conditions (67–74°F and 30–70%).

### Ex vivo Analysis

At the end point, tumors were harvested, weighed and minced up. Total protein from frozen tissues was prepared with T-PER (ThermoFisher Scientific) supplemented with protease and phophatase inhibitors (Roche). Equal quantities of proteins were combined with 5X protein loading buffer and separated by SDS-PAGE followed by PVDF membrane transfer. Membranes were blocked with 5% milk followed by incubation with LC3 (Cell Signaling) and GAPDH (ThermoFisher) antibodies. Blots were developed with ECL Reagents (Pierce).

### Maximum tolerated dose (MTD) Studies

MTD studies were outsourced and performed at a local CRO based on their established protocols.

### hERG assay

The fluorescence polarization-based assay to characterize the affinity of 249C for human *ether-a-go-go*-related gene (hERG) responsible for electrical activity of the heart was performed as previously described^90^.

### BioMAP

The BioMAP Toxicity Screening Profile was performed as described previously^91^.

### Kinome scan

The KINOMEscan™ Profiling Service consisting of 468 human kinases was used to assess 249C inhibitor activity to check for off-target effects as previously described^92^.

### LDH Assay

Cells were left untreated (DMSO) or treated with indicated concentrations of 249C, 68, 148D, 226C for 48 hours. The LDH Cytotoxicity assays were performed using a kit (Pierce 88953) and following the manufacturer’s recommendations.

### Statistical analyses

All experiments were repeated a minimum of two times with at least duplicate or triplicate samples and measurements were taken from distinct samples. Values including controls are expressed as the mean ± stdv *or* mean ± sem. Two-tailed paired Student’s *t* test was used to test for differences between two groups unless indicated otherwise. Differences with a p≤0.05 were considered as statistically significant.

## Supplementary Material

Supplementary Info

Source Data for Fig 2

Source Data for Fig 4

Supplementary Tables

## Data Availability

Datasets that support the findings of this study are available in Source Data/Supplementary Figures and Tables. Mutation Analysis in CCLE Cell Lines: The Combined Annotation Dependent Database (https://cadd.gs.washington.edu/) was used to assess the predicted functional importance of SNVs (version 1.6). Cell line mutation data (CCLE_DepMap_18q3_maf_20180718.txt) were downloaded from the Cancer Cell Line Encyclopedia (CCLE) at https://depmap.org/portal/download/ (2018). Mass Spectrometry GO gene sets: GSEA (version 4.2.3) software and Molecular Signature Database (MSigDB version 7.4) from http://www.broad.mit.edu/gsea was used to query the top proteins from the Whole Proteome Analysis for GO gene sets in NCI-60 cell lines. CRISPR Screen: sgRNA read counts and phenotypes for all pooled screens are provided as supplementary tables. All other data will be made available by the corresponding author upon request.
