# Supplementary material for: Ras-mutant cancers are sensitive to small molecule inhibition of V-type ATPases in mice
Source: Nat Biotechnol. Author manuscript; Available in PMC 2022 Dec 22. (PMC9750872; doi:10.1038/s41587-022-01386-z)
Supplement: Source Data for Fig 2 [file NIHMS1826099-supplement-Source_Data_for_Fig_2.pdf]

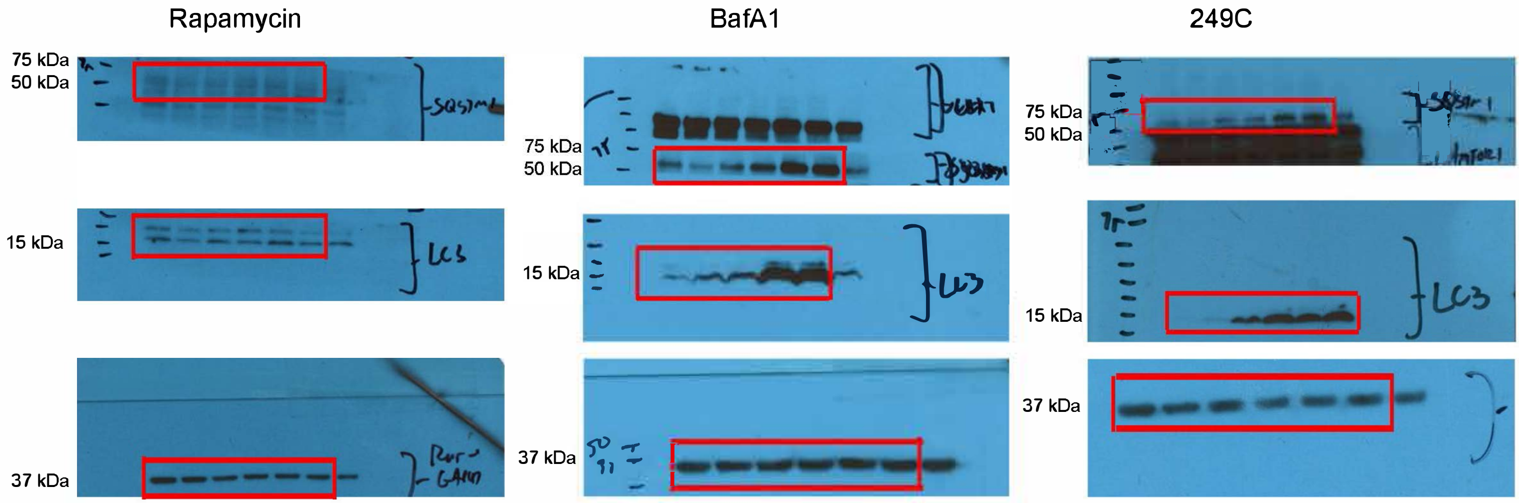

Source Data for Fig. 2: Full-length Western blots for A549 cells treated with molecules (Rapamycin, BafA1, and 249C). DMSO, 2h, 4h, 8h, 20h, 24h. Blotted for SQSTM1 (62 kDa), LC3-I/II (~14-16 kDa), and GAPDH (~37 kDa)
