# Supplementary material for: Ras-mutant cancers are sensitive to small molecule inhibition of V-type ATPases in mice
Source: Nat Biotechnol. Author manuscript; Available in PMC 2022 Dec 22. (PMC9750872; doi:10.1038/s41587-022-01386-z)
Supplement: Source Data for Fig 4 [file NIHMS1826099-supplement-Source_Data_for_Fig_4.pdf]

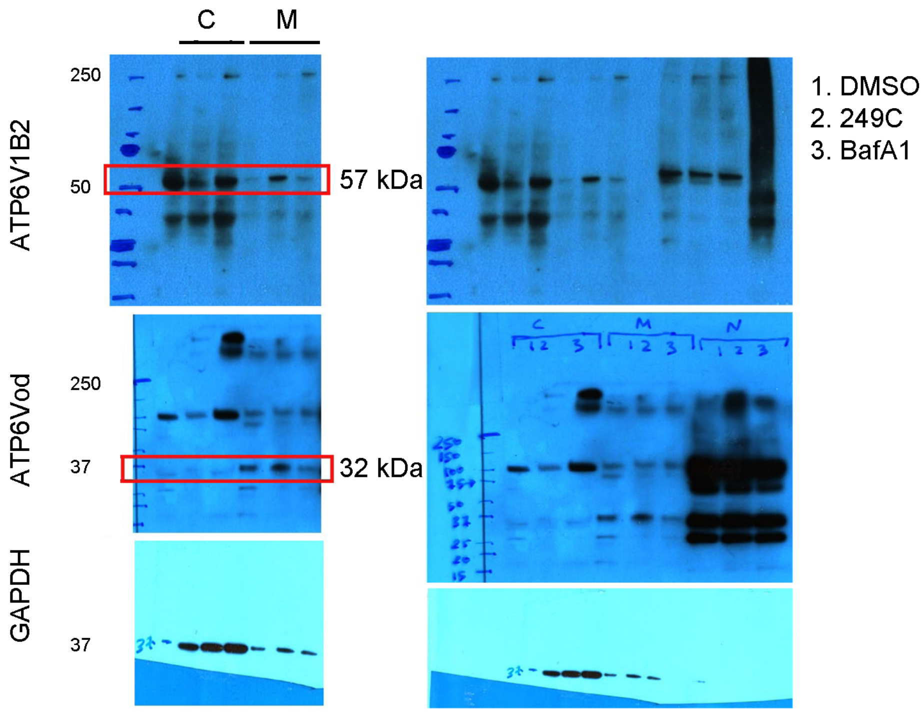

Source Data for Fig. 4: Cropped (left panel) and corresponding uncropped (right panel) Western blots. C = cytoplasmic, M = membrane fractions. 1 = DMSO, 2 = 249C, 3 = BafA1. Blotted for ATP6V$_1$B2 (57 kDa), ATP6V0d 32 kDa), and GAPDH (~37 kDa)
