# Supplementary material for: Ras-mutant cancers are sensitive to small molecule inhibition of V-type ATPases in mice
Source: Nat Biotechnol. 2022 Jul 25;40(12):1834–44. doi: 10.1038/s41587-022-01386-z (PMC9750872; doi:10.1038/s41587-022-01386-z)
Supplement: Supplementary file 1 [file 41587_2022_1386_MOESM1_ESM.pdf]

nature portfolio

https://doi.org/10.1038/s41587-022-01386-z

Supplementary information

Ras-mutant cancers are sensitive to small molecule inhibition of V-type ATPases in mice

In the format provided by the authors and unedited

Tolani Supplementary Figures

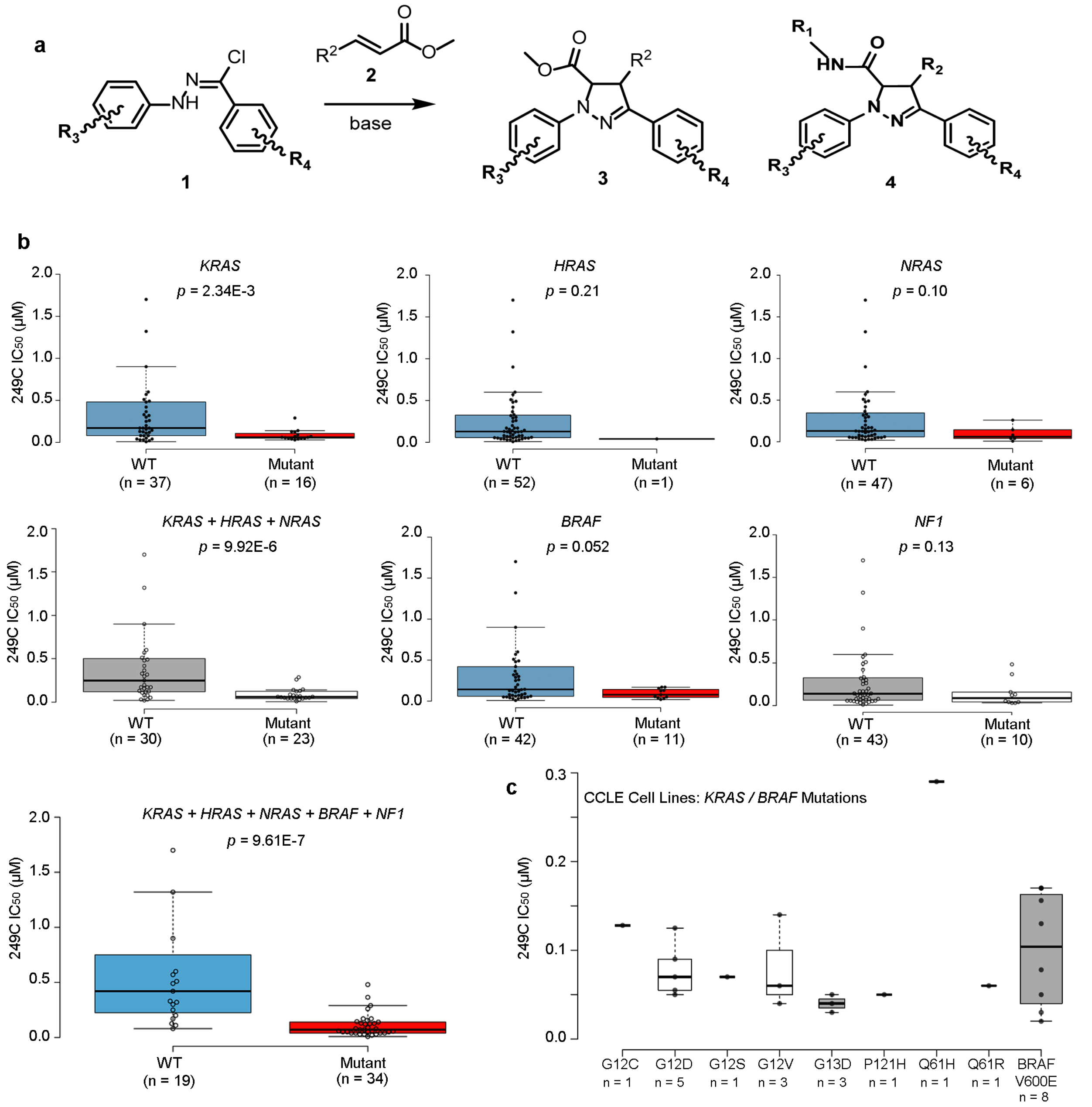

Supplementary Figure 1: General Synthesis Scheme for Preparation of Compound Library and Mutant Gene Associations with 249C IC_{50} Values. (**A**) Rationally designed small molecules to represent diversity in the compound library were synthesized by the synthesis method depicted. The compound depicted by the general structure **3** can be synthesized from a,b-unsaturated esters (**2**) and hydrazonyl chlorides (**1**) in a [3+2] cycloaddition reaction. Subsequent deprotection of the methyl ester (**3**) yields free carboxylic acid, amenable for final compound (**4**) generation *via* amide bond formation. (**B**) 249C IC_{50} values in 53 cancer cell lines stratified by the mutation status of: 1) *KRAS;* 2) *HRAS;* 3) *NRAS*; 4) *KRAS+HRAS+NRAS;* 5) *BRAF;* 6) *NF1;* and 7) *KRAS+HRAS+NRAS+BRAF+NF1* (two-sided Wilcoxon rank sum tests). (**C**) 249C IC_{50} values in these cancer cell lines (Cancer Cell Line Encyclopedia) from Fig. 1e stratified by the mutation status of *KRAS/BRAF.* In a Wilcoxon rank sum test comparing IC_{50} values of the three *KRAS*G13D mutant cell lines versus IC_{50} values of the non-G13D mutant cell lines, IC_{50} is significantly lower for G13D (p <
represents the median, the upper and lower bounds of the box indicate the interquartile range (IQR, the range between the 25th and 75th percentiles), and whiskers extend to the highest and lowest values within 1.5 times the IQR.

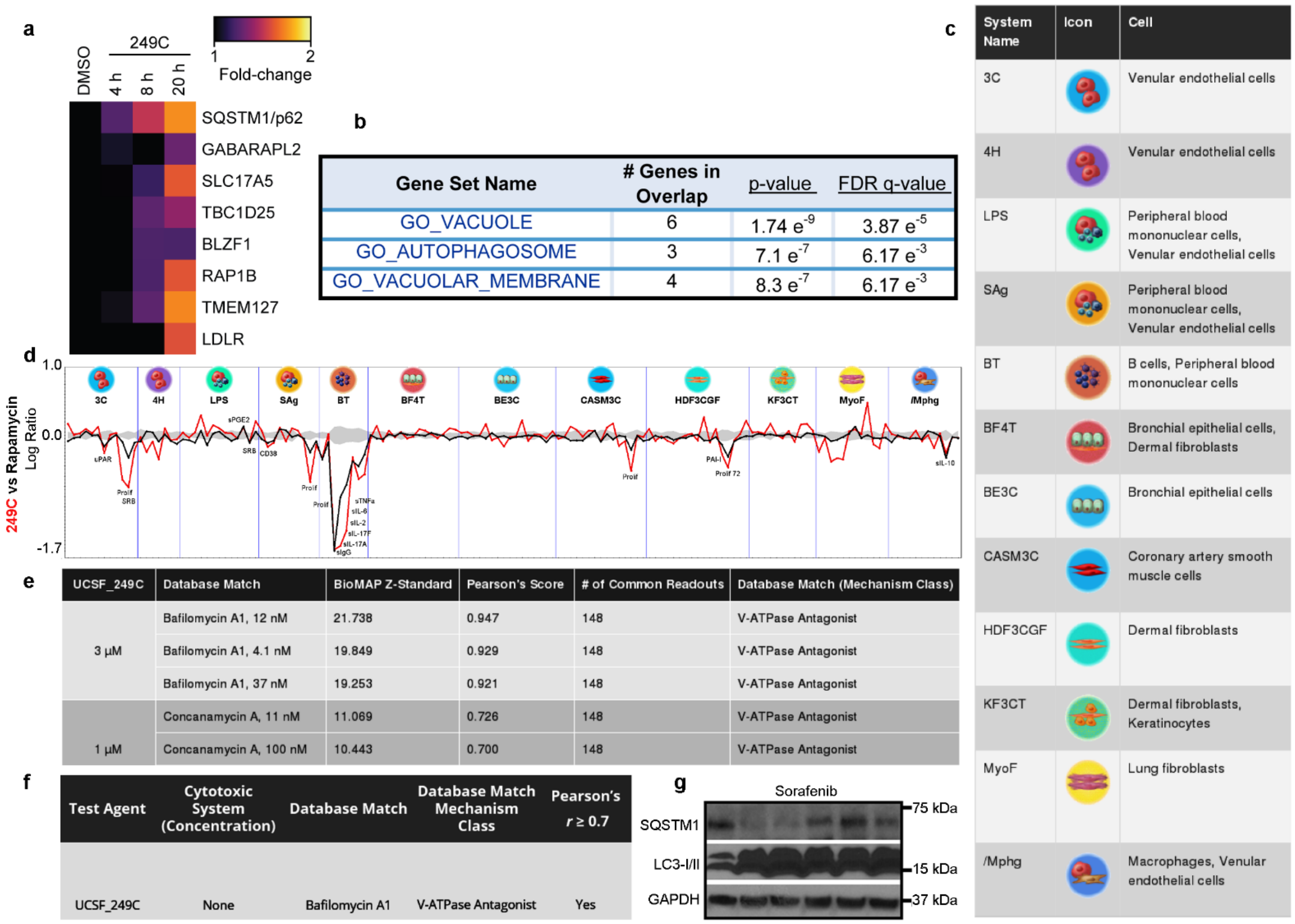

Supplementary Figure 2: Top BioSeek Reference Database Matches for 249C. (**A**) Quantitative whole proteome analysis of the top hits in 249C-treated A549 cells over time relative to DMSO control. (**B**) GSEA MsigDB query results from proteins in (a) reveals perturbations in the autophagy pathway. A one-sided hypergeometric test inbuilt into the online software was used to calculate *p*-values. (**C**) Table of the 12 cell-based systems used to query 249C, including a list of cell types. (**D**) Overlay of **249C** vs **Rapamycin** (used as a reference benchmark), an inhibitor of the mTOR pathway. (**E**) Top reference database matches for 249C from an unsupervised search of >4,000 agents. The similarity between agents is determined using a combinatorial approach that profiles and ranks (BioMAP Z-Standard) the Pearson's correlation coefficient between two profiles. If the Pearson's correlation ≥ 0.7, profiles are identified as having mechanistic similarity. (**F**) Summary of test agent 249C. (**G**) Similar to 249C, treatment with autophagy modulator Sorafenib results in upregulation of autophagy markers SQSTM1/p62 and LC3-I/II over time (DMSO, 2h, 4h, 8h, 20h, 24h). Representative of 3 independent experiments.

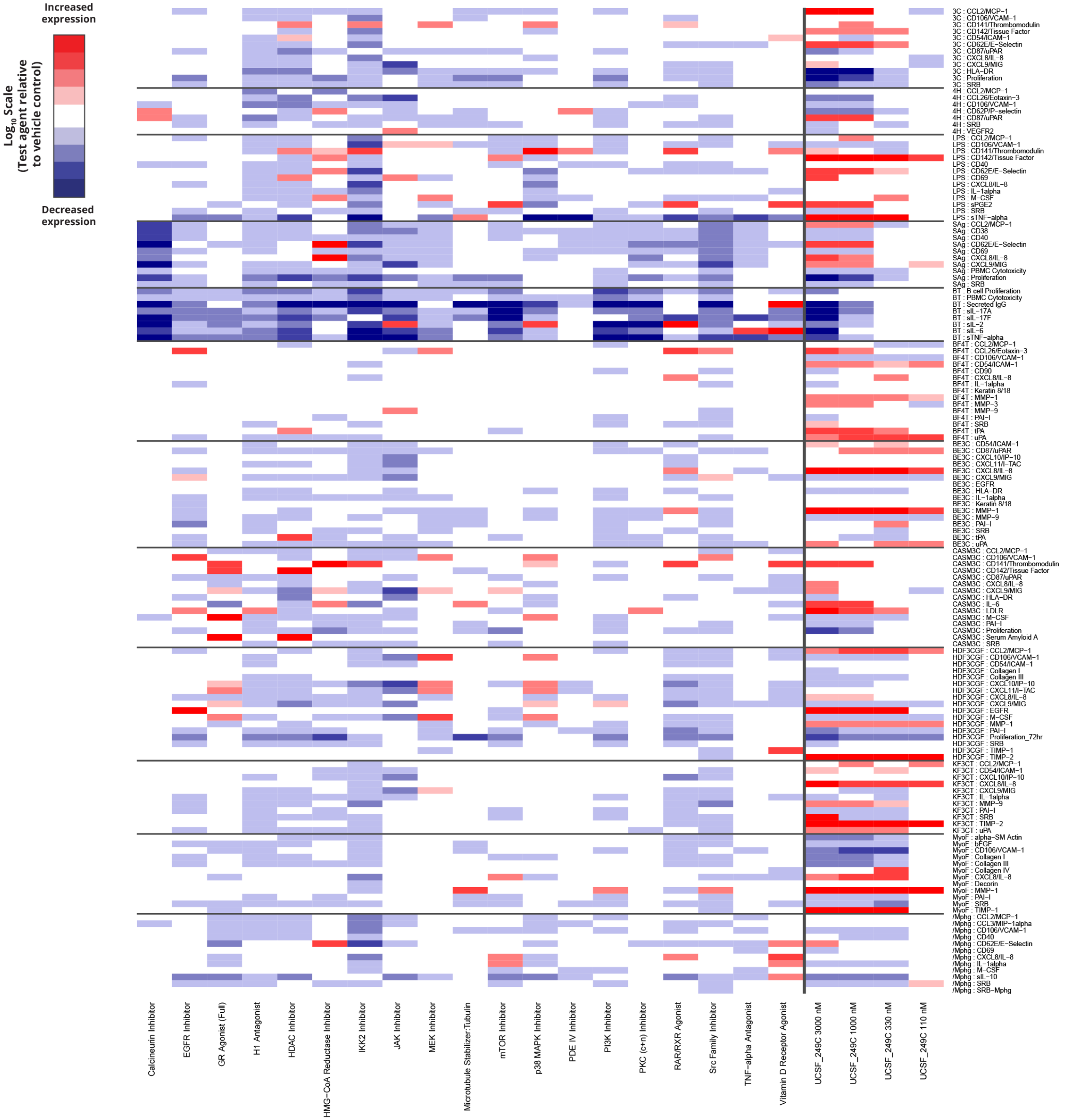

Supplementary Figure 3: Mechanism Heatmap Analysis of 249C. Heatmap analysis of the 148 biomarker readouts by 249C in comparison to 19 consensus mechanism class profiles (columns). These profiles were generated from a larger reference database[7,8].

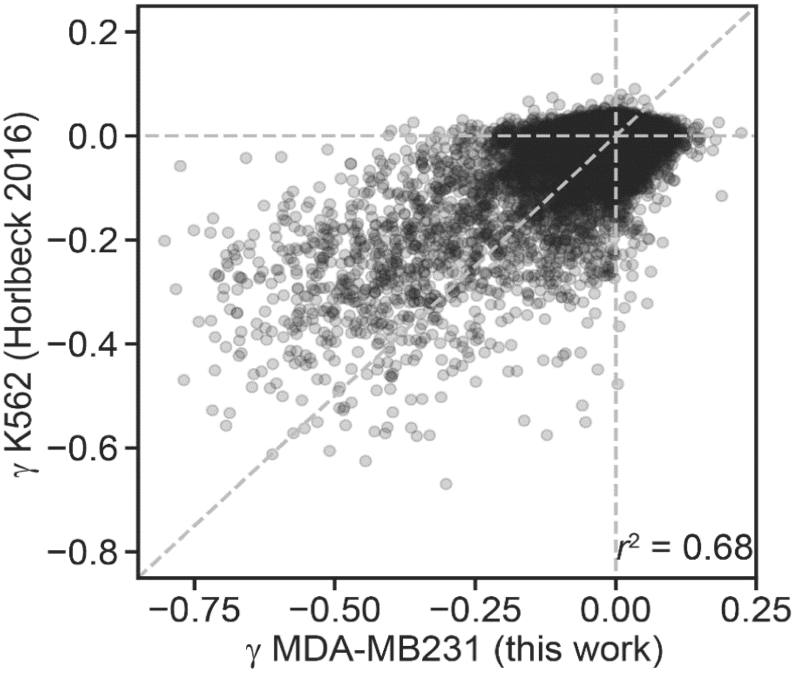

Supplementary Figure 4: Comparison of Untreated Growth Phenotypes in MDA-MB-231 and K562 Cells. Untreated growth phenotypes for K562 cells are derived from ref 25. r^2 is the squared Pearson correlation coefficient.

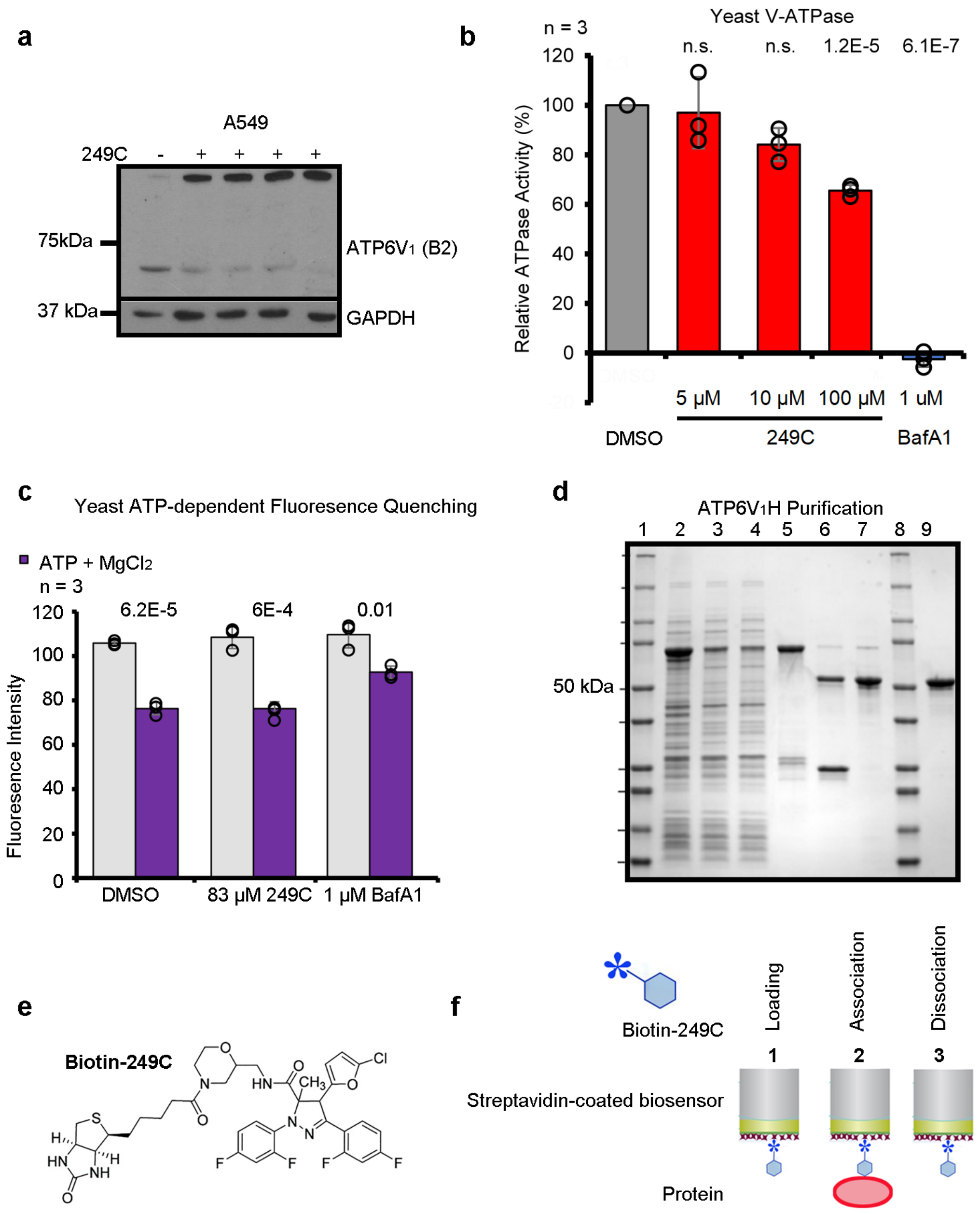

Supplementary Figure 5: Biochemical and Biophysical Effects of 249C on V-ATPase. (**A**) Immunoblot of A549 cells treated with 249C over time (2h, 4h, 8h, 20h) tested for ATP6V1B2 (~54 kDa). Molecule treatment appears to cause accumulation of high molecular weight entities. Representative of 3 independent experiments. (**B**) Yeast V-ATPase activity measured in the absence and presence of 249C and BafA1; only modest inhibition observed. Data shown are mean ± SD (n = 3) Two-tailed student's *t*-test (**C**) ATP-dependent fluorescence quenching in yeast (mean ± SD; n = 3) Two-tailed student's *t*-test (**D**) SDS PAGE over the course of recombinant human ATP6V$_1$H protein purification. 1. Markers; 2. Total lysate (GST-fusion 70.5 kDa); 3. Soluble lysate; 4. GSTrap flow-through; 5. GSTrap elute; 6. Post thrombin cleavage (target 54 kDa, GST 25 kDa); 7. Subtractive GSTrap flow-through; 8. Markers; 9. Final sample, 1 µg protein load. Representative of 2 independent experiments. (**E**) Structure of biotin-249C. Addition of the biotin-linker at the R1 position does not appear to interfere with V-ATPase binding. (**F**) Experimental schematic of Bio-layer inferometry (BLI).

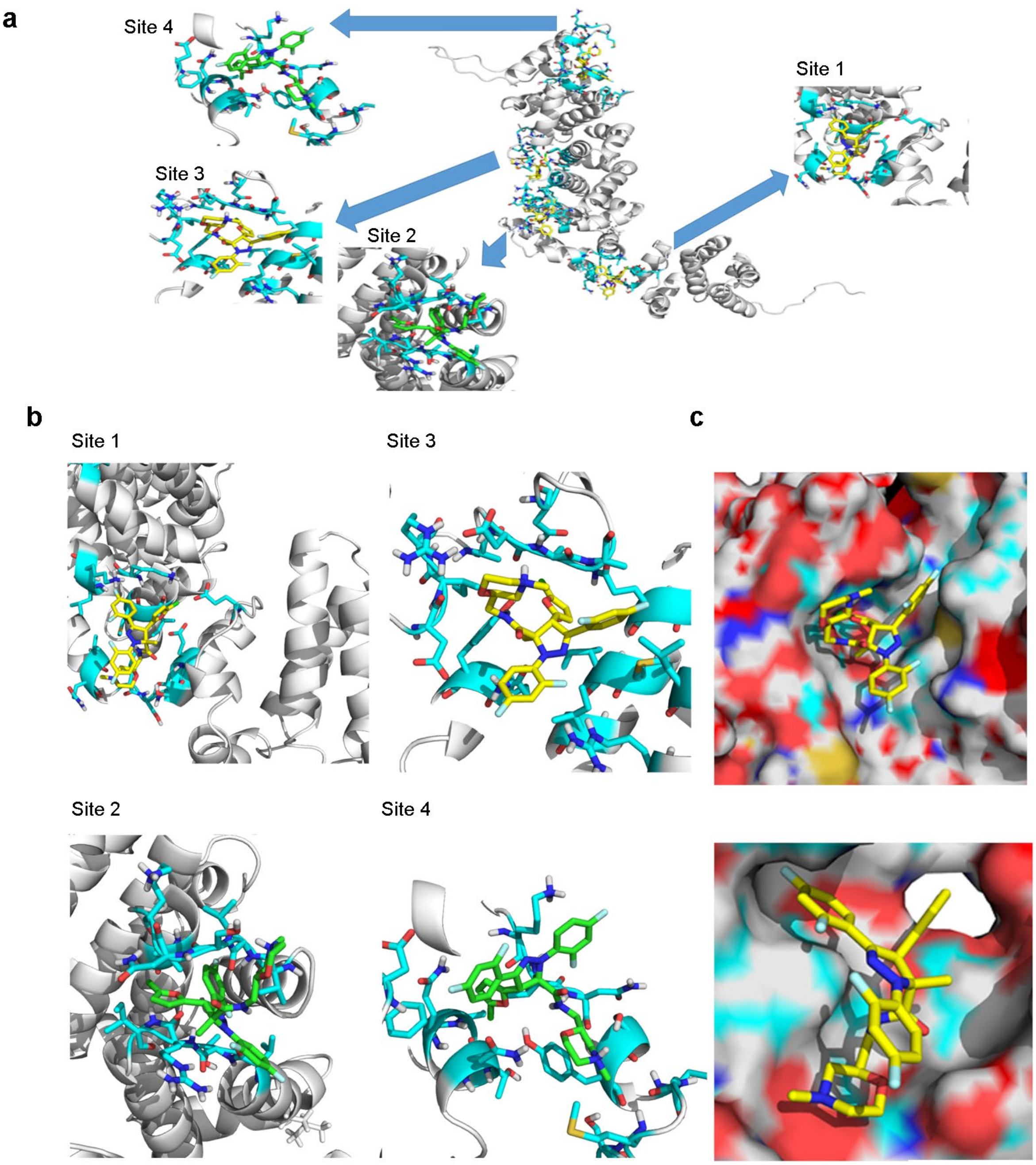

Supplementary Figure 6: 3D Molecular Modeling, Binding Pocket Detection and Docking. (**A**) 4 predicted docking sites for 249C against the H subunit (ATP6V$_1$H) based on the crystallized form in yeast (PDB ID 1HO8). At the time this study was performed, the closest sequence analogue was the yeast isoform (uniprot # P41807), which shares 27% identity and 45% sequence similarity, making it a feasible template for modeling the human protein. (**B**) Common features between all these binding sites are packing and aromatic interactions between the protein and the furane ring, as well as one of the phenyl rings. The other phenyl group and the morpholine rings are exposed to the solvent. (**C**) Among these 4 predicted binding sites, 2 are more buried than the others, as shown by the surface representation. In both cases, the morpholine amine can interact with carbonyl groups within the protein binding site (red surfaces). It is possible that allowing the binding site to relax will favor a more buried conformation of the protein, improving the interaction landscape. Currently we cannot assess if the putative binding site is conserved across species since we detected 4 possible sites. Despite the high sequence similarity reliable enough to make a homology model, the sequence identity is fairly low, which would affect the protein ligand-interaction landscape.

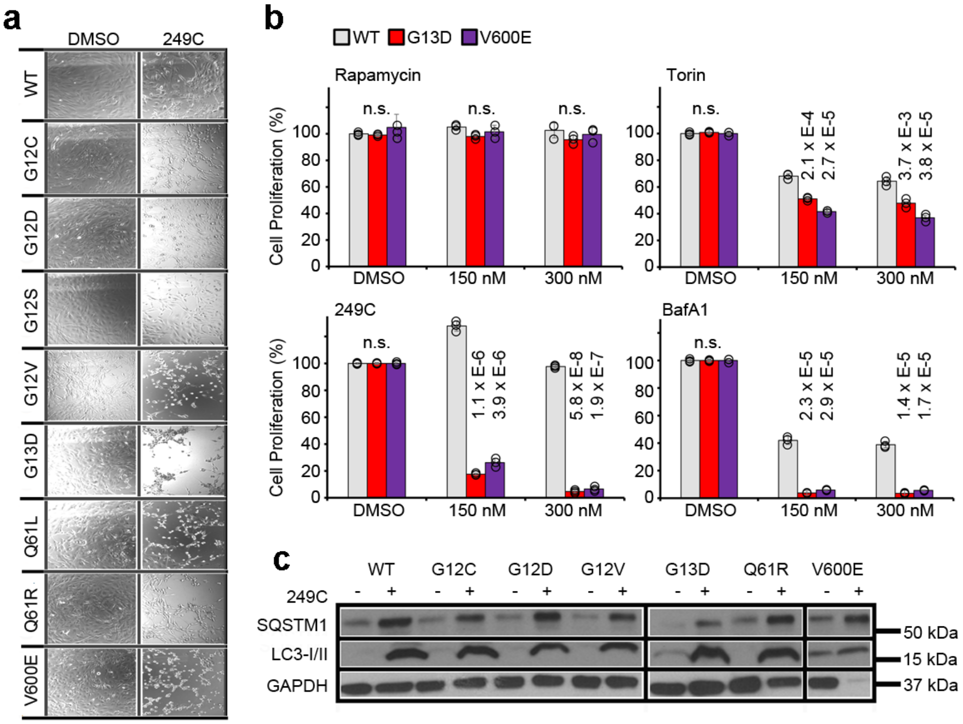

Supplementary Figure 7: 249C Treatment Differentially Affects Cells Bearing Mutations in *KRAS* and *BRAF*. (**A**) Bright field images of immortalized MEFs obtained from the NCI harboring single point mutations in *KRAS* and *BRAF* treated with 249C. Images were captured at 20X. (**B**) Cell proliferation assays of murine fibroblasts bearing single point mutations in human ***KRAS*G13D** or ***BRAF*V600E** compared to wild type ***KRAS*WT** post treatment with autophagy inducers: Rapamycin and Torin, and autophagy inhibitors: 249C and BafA1. 249C is less toxic on WT cells relative to BafA1. Representative of 2 independent experiments. Data shown are mean ± SD; n = 3. Two-sided *t*-test with no adjustments made for multiple comparisons. (**C**) Immunoblot analyses of 249C-treated (400 nM) cells show an up-regulation of SQSTM1 and LC3-I/II expression when compared with the DMSO controls in MEFs. Representative of 3 independent experiments.

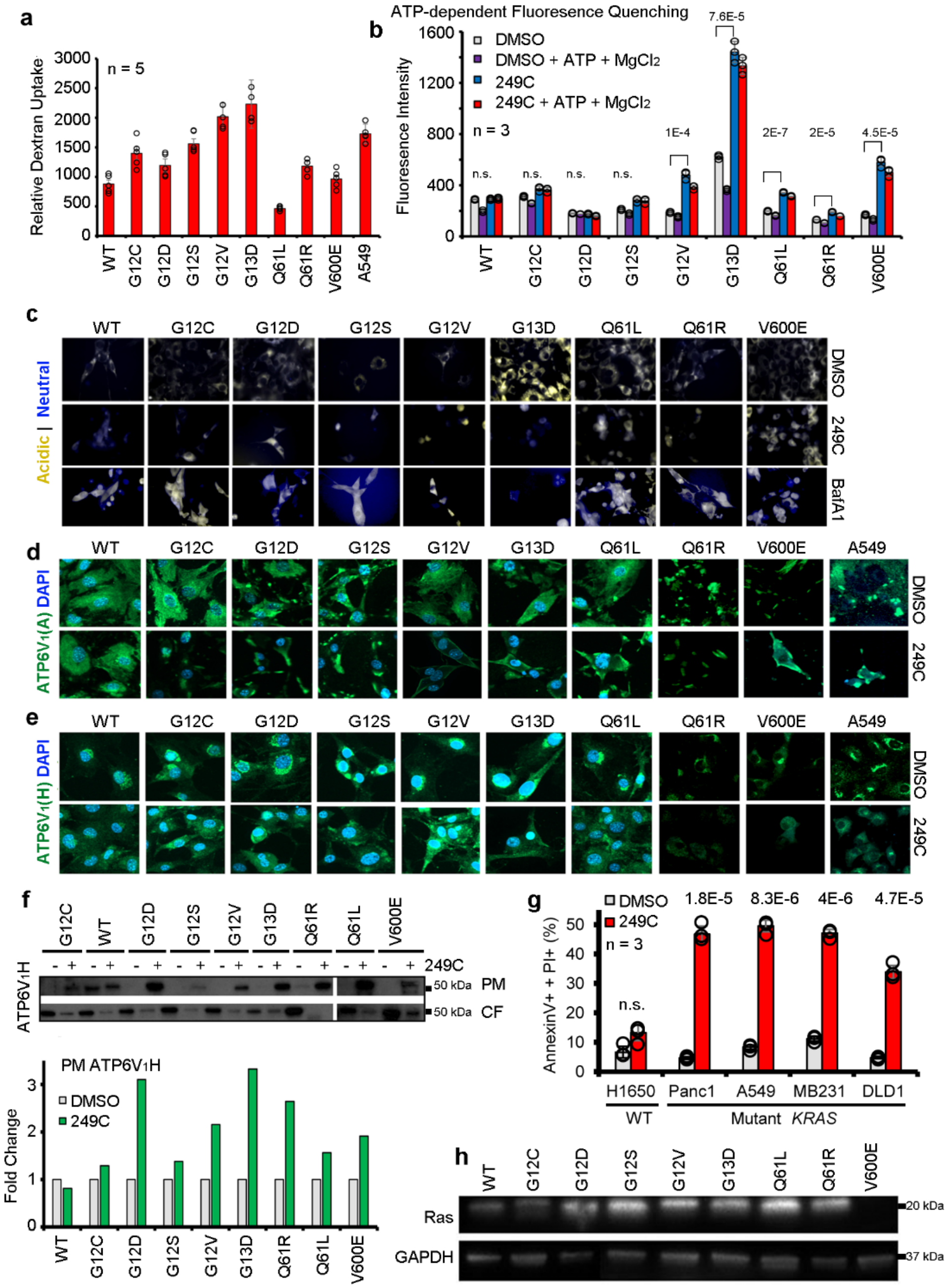

Supplementary Figure 8: Effect of 249C Treatment *in vitro*. (**A**) Quantification of TMR–dextran uptake in MEFs. *KRAS*G12V and G13D exhibit the highest basal levels of macropinocytosis. Representative of 3 biological replicates, n = 5; data are mean ± SD. (**B**) ATP-dependent fluorescence quenching in MEFs after 2 h of 249C treatment (or DMSO) as in Fig. 3f. mean ± SD Representative of 2 individual experiments with n = 3, for each is presented. Two-tailed student's *t*-test (**C**) Treatment with autophagy inhibitors, 249C and BafA1, resulted in changes in pH (yellow: acidic; blue: neutral | LysoSensor) relative to the DMSO control in MEFs. Representative images from n >3. (**D**) Effect of 249C treatment on V-ATPase localization in MEFs. of MEFs bearing WT and mutant *KRAS* cells immunostained for V1A subunit and V1H subunit. (**E**); >3 images were captured per well. Representative of 2 biological replicates presented. (**F**) Immunoblots of ATP6V$_1$H in the plasma membrane (PM) and cytoplasmic fraction (CF) of MEFs (top panel) and fold change quantification (bottom panel) in PM ATP6V1H using ImageJ. Representative of 2 biological replicates. (**G**) Treatment with 100 nM 249C induces cell death more strongly in *KRAS* mutant cancer cells, A549 (lung), MB-231 (breast) and DLD1 (colorectal) relative to *KRAS* WT cells, H1650 (lung), assessed by quantification of double positive Annexin-V$^+$/PI$^+$ cells by flow cytometry at 48 hours relative to DMSO controls. Data shown are mean ± SD (n = 3); Two-tailed student's *t*-test (**K**) Western blot for Ras in the different MEF lines.

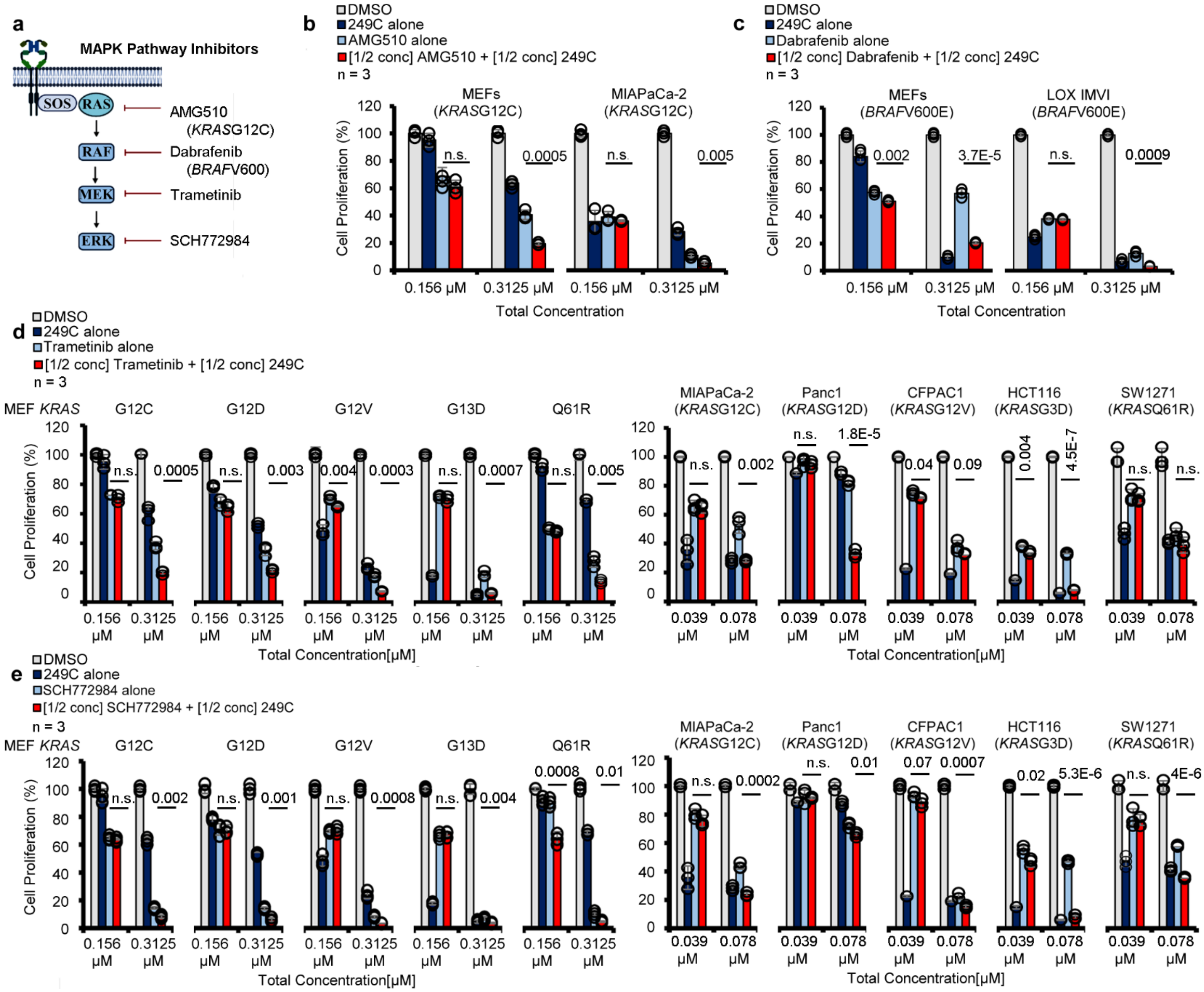

Supplementary Figure 9: 249C Sensitizes Cells in Combination with MAPK Pathway Inhibitors. (**A**) The following MAPK pathway inhibitors were used in combination with 249C: AMG510 (*KRAS*G12C), Dabrafenib (*BRAF*V600), Trametinib (MEK1/2) and SCH772984 (ERK1/2) (**B**) Cell proliferation of *KRAS*G12C bearing cells [MEFs (fibroblasts) and MIAPaCa-2 (cancer cell line)] co-treated with AMG510 (*KRAS*G12C inhibitor) and 249C (V-ATPase inhibitor) at 72 hours. For combination treatments, one half of the concentration of each of the molecules was combined, such that the final dose equaled to the single drug alone to facilitate easy comparisons. For single dose 249C data, refer to Figures 1 and 4. (**C**) Cell proliferation of *BRAF*V600E bearing cells [MEFs (fibroblasts) and LOX-IMV1 (cancer cell line)] co-treated with Dabrafenib (*BRAF*V600 inhibitor) and 249C (V-ATPase inhibitor). (**D**) Cell proliferation of select MEFs (*KRAS*G12C, G12D, G12V, G13D, and Q61R) and cancer cell lines [MIAPaCa-2 (*KRAS*G12C), Panc1 (*KRAS*G12D), CFPAC1 (*KRAS*G12V), HCT116 (*KRAS*G13D), and SW1271 (*KRAS*Q61R)] co-treated with Trametinib (MEK1/2 inhibitor) and 249C. (**E**) Cell proliferation of select MEFs (*KRAS*G12C, G12D, G12V, G13D, and Q61R) and cancer cell lines [MIAPaCa-2 (*KRAS*G12C), Panc1 (*KRAS*G12D), CFPAC1 (*KRAS*G12V), HCT116 (*KRAS*G13D), and SW1271 (*KRAS*Q61R)] co-treated with SCH772984 (ERK1/2 inhibitor) and 249C. Two-tailed student's *t*-test. Data shown are mean ± SD; n = 3 for all. Note: Cancer cell lines are more sensitive to 249C than MEFs and thus a lower dose of the molecule was used for them. All data are representative of three independent experiments.

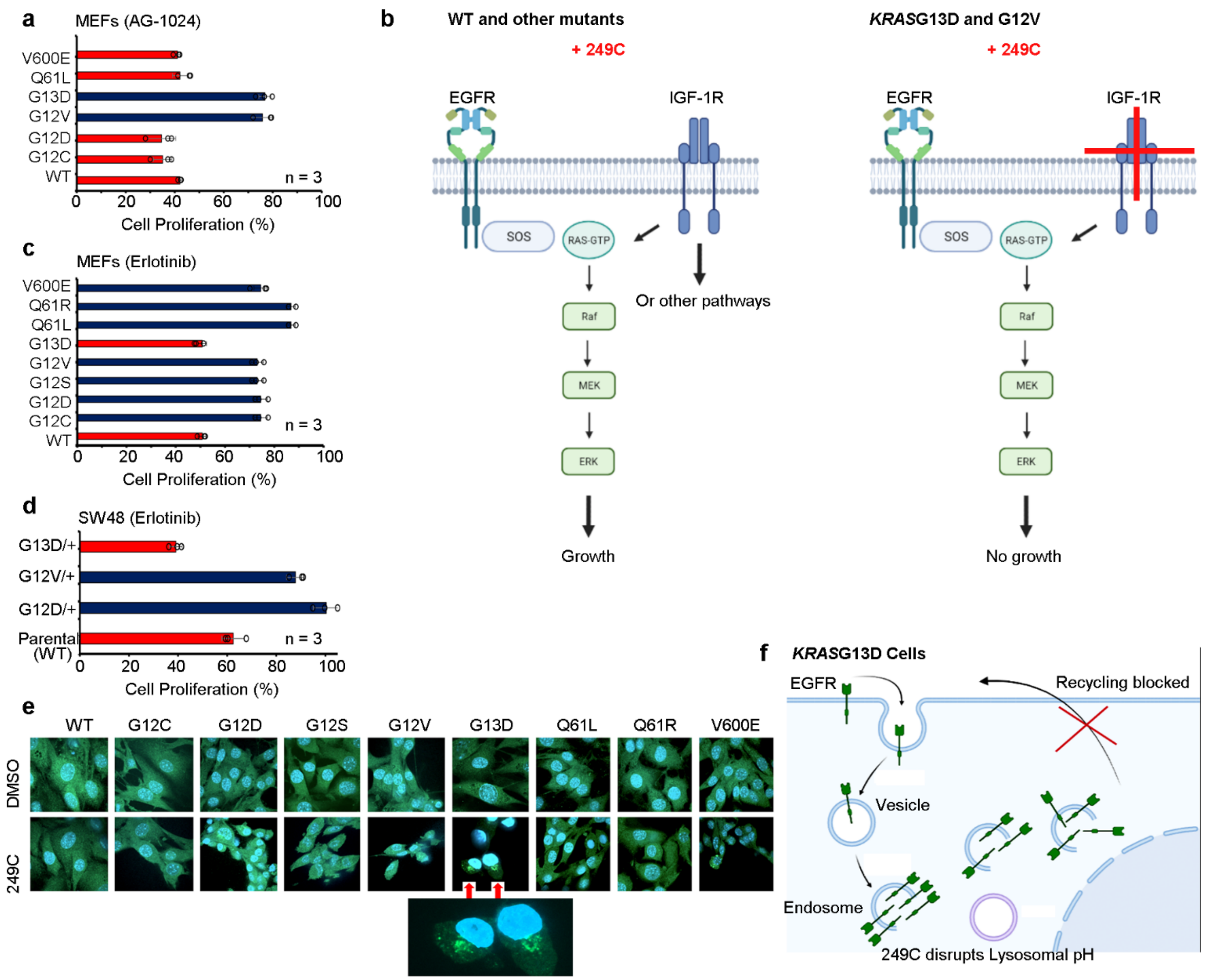

Supplementary Figure 10: *KRAS* Mutants G12V and G13D Exhibit Unique Signaling. (**A**) Cell proliferation of MEFs with IGF-1R inhibitor AG-1024 (10 µM). (**B**) Schematic for mutant specific *KRAS* signaling. In the presence of 249C, mutants G12V and G13D are unable to signal via IGF-1R but WT and other mutants can (and possibly via additional pathways as well). (**C**) Cell proliferation assays of MEFs with EGFR inhibitor Erlotinib (5 µM). Data shown are mean ± SD; n = 3 for all. (**D**) Cell proliferation of SW48 cells with Erlotinib (40 nM). G13D and WT cells in both MEFs and SW48 models responded to Erlotinib treatment indicating their dependence on EGFR signaling but other mutants were not as responsive. (**E**) Effect of 249C treatment on EGFR localization in MEFs. Fluorescence micrographs of MEFs bearing WT and mutant *KRAS* cells immunostained for EGFR; >3 images were captured per well. Representative of 2 biological replicates presented. (**F**) Schematic of EGFR trafficking in *KRAS*G13D cells in the presence of 249C.

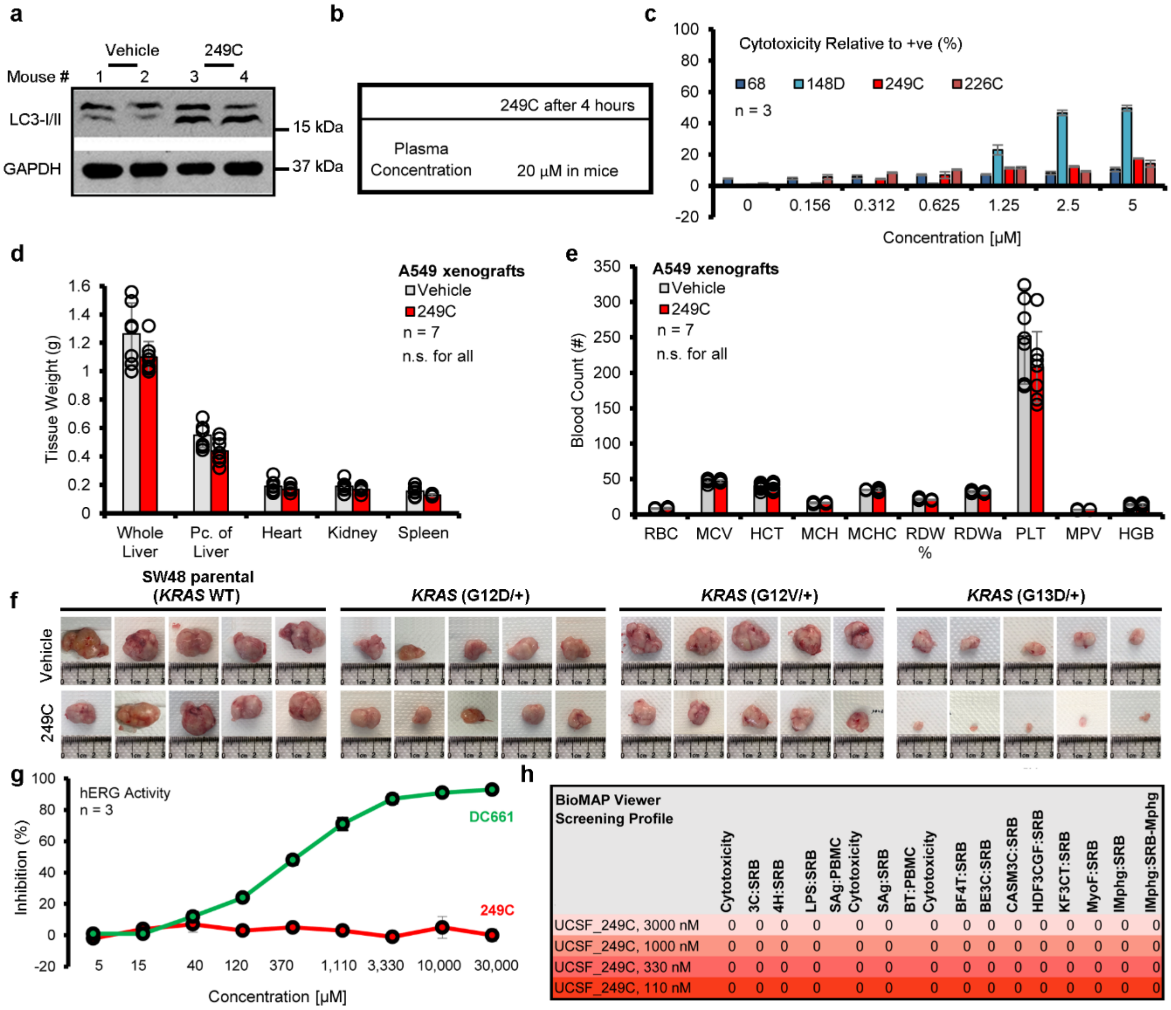

Supplementary Figure 11: *Ex vivo*, *in vivo* and Safety Profile for 249C. (**A**) *Ex vivo* analyses of 249C-treated tumors show an up-regulation of LC3-I/II expression when compared with the DMSO controls in A549 lung cancer xenografts. Representative of 2 independent experiments. (**B**) After 4 hours, a safe maximum concentration (C_{max}) of 20 µM of 249C was detected in blood (or plasma) of mice. (**C**) Cytotoxicity mediated by molecules 68, 148D, 249C and 226C relative to a cytotoxic positive control. Lactate dehydrogenase (LDH) is a cytosolic enzyme released upon plasma membrane damage into the culture media which is quantified colorimetrically. Data shown are mean ± SD; n = 3. Representative of 2 biological replicates. (**D**) No significant difference in tissue weight or (**E**) the number of cellular counts in the blood (mean ± SD; n = 7) harvested from mice bearing A549 xenografts treated with 249C or the vehicle control (mean ± SD; n = 7) from Figure 6a. Two-tailed student's *t*-test (**F**) *in vivo* antitumor activity of 249C on tumor volume in isogenic xenografts: SW48 parental (*KRAS*WT), SW48 *KRAS*G12D/+, SW48 *KRAS*G12V/+ and SW48 *KRAS*G13D/+ at Day 21 after 14 days of treatment (10 mg/kg 249C or vehicle control, i.p., n = 5 for all arms). (**G**) Percent inhibition in fluorescence polarization of human *ether-à-go-go*-related gene (hERG) responsible for electrical activity of the heart by **DC661** (100% inhibition at 30 µM) and **249C** (0% inhibition at 30 µM) (mean ± SD; n = 3). (**H**) *in vitro* BioMap® Safety and Toxicology screening profile of 249C reveals no adverse events at 4 doses across over 100 biomarker readouts (see methods for additional details).

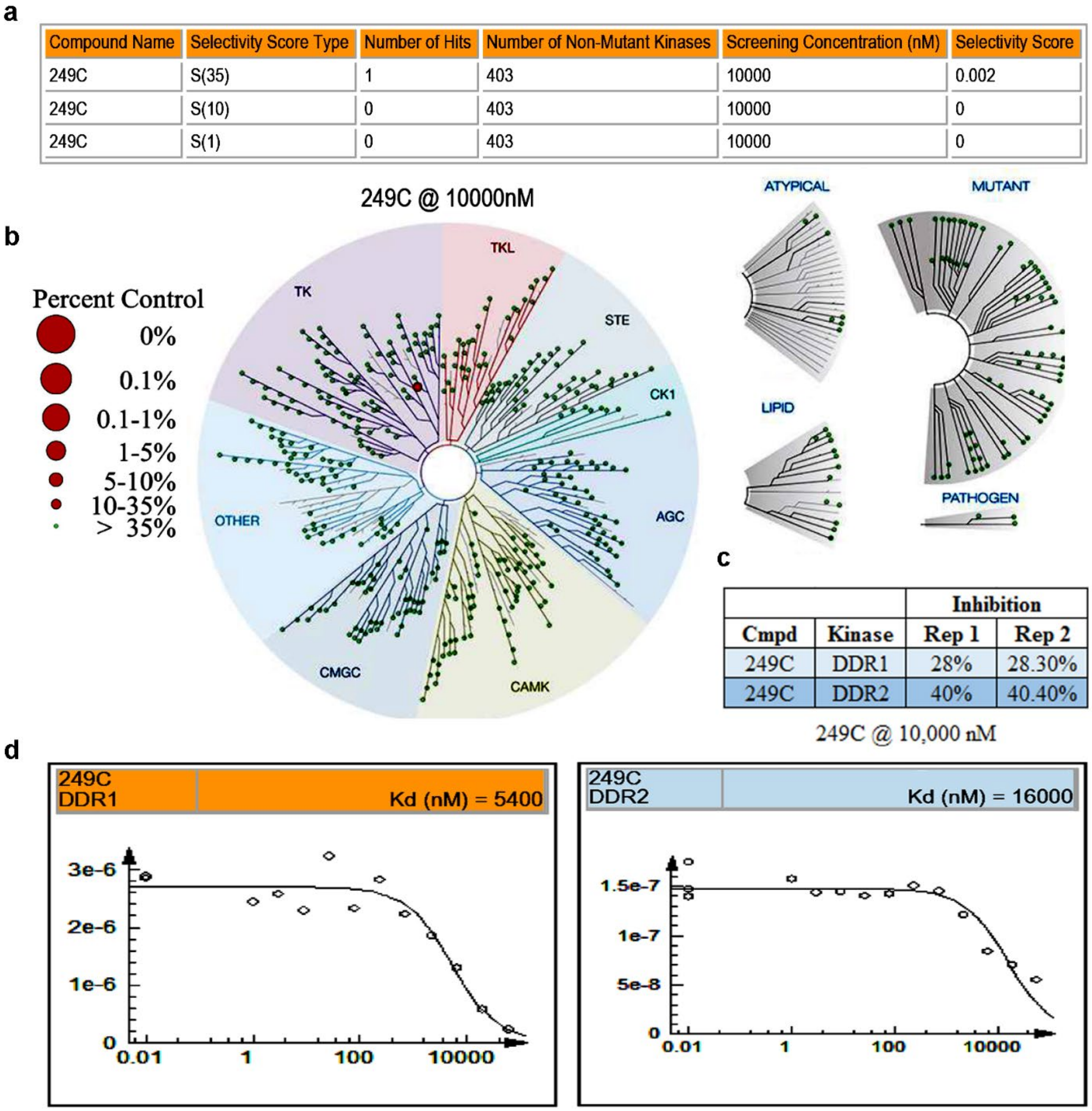

a

Compound Name	Selectivity Score Type	Number of Hits	Number of Non-Mutant Kinases	Screening Concentration (nM)	Selectivity Score
249C	S(35)	1	403	10000	0.002
249C	S(10)	0	403	10000	0
249C	S(1)	0	403	10000	0

c

		Inhibition	
Cmpd	Kinase	Rep 1	Rep 2
249C	DDR1	28%	28.30%
249C	DDR2	40%	40.40%

249C @ 10,000 nM

Supplementary Figure 12: Compound 249C Assessed for off-target Effects using the KINOME*scan*™ Screening Platform. (**A**) Selectivity (S-score) for 249C where the score is a quantitative measure of compound selectivity against human kinases. It is calculate by dividing the number of kinases that compounds bind to by the total number of distinct kinases tested, excluding mutant variants (S = number of hits / number of assays). 1 hit out of >400 kinases is reported for test compound (249C) = 0.002. S(35) = number of non-mutant kinases with % ctrl <35/number of non-mutant kinases; (**B**) Kinome interaction map for 249C at 10,000 nM. Each red dot indicates a kinase found to bind, where larger dots indicate higher affinity interactions. 249C was found to bind 1 Tyrosine Kinase (TK) out of >400 kinases (created in TREE*spot*™) with low affinity. This is calculated using %Ctrl as a potency threshold [negative control = DMSO (100% Ctrl) and positive control = control compound (0% Ctrl)]. (**C**) Re-screen of TKs DDR1 and DDR2 indicate modest inhibition with 249C at 10,000 nM. (**D**) An 11-point 30-fold serial dilution of 249C against DDR1/2. Dissociation constants (K_{ds}) were determined using 249C at 30,000 nM, Hill slopes were set to -1 and curves were fitted using a non-linear least square fit with the Levenberg-Marquardt algorithm; K_d values: DDR1 = 5,400 nM and DDR2 = 16,000 nM

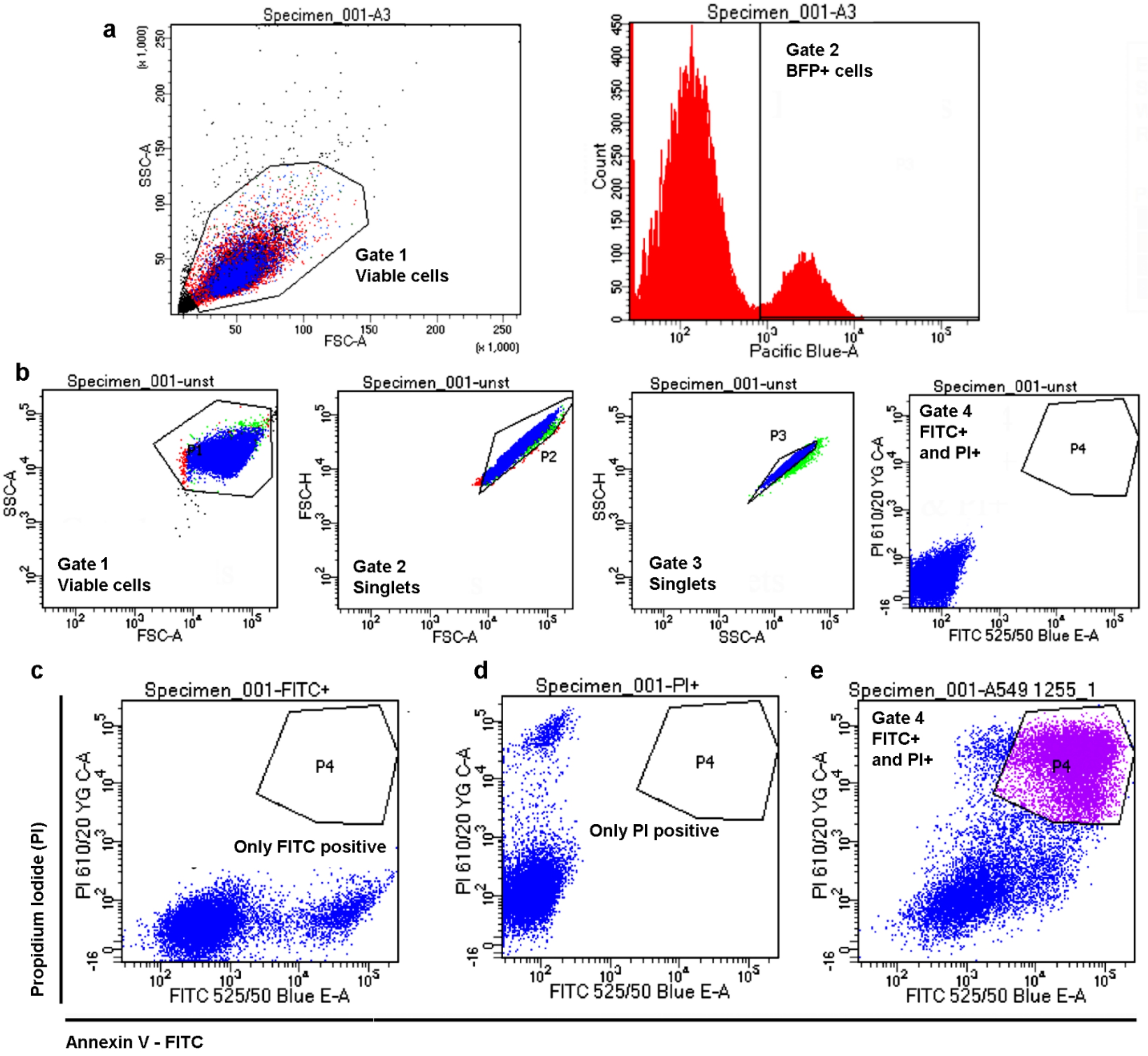

Supplementary Figure 13: (**A**) Representative plots illustrating gating strategy to select blue fluorescent protein (BFP) positive (+) cells for analysis of the individual CRISPR sgRNA re-tests in Figure 3. Gate 2 demarcates BFP^+ cells. (**B**) Representative plots illustrating gating strategy for the apoptosis assays to quantify double positive cells (AnnexinV-FITC and Propidium Iodide (PI)) in Figure 5. Gate 1 demarcates viable cells without any stain. Gates 2 and 3 exclude doublets for the same population. Gate 4 sets the gate for $FITC^+PI^+$ cells showing no positive cells for the unstained population (control). Single color controls: Cells only stained with FITC (**C**) show a shift on the x-axis ($FITC^+$) and cells only stained with PI (**D**) show a shift on the y-axis (PI^+), and cells treated with 249C (1255) (**E**) show a double positive population ($FITC^+PI^+$) bound by Gate 4.

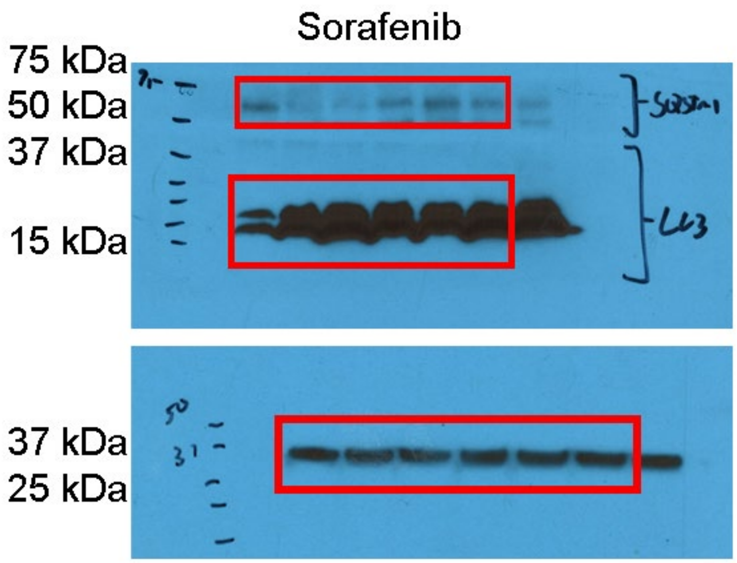

Supplementary Figure 14: Full-length Western blots for Supplementary Fig 2g. A549 cells were left untreated (DMSO) or treated with Sorafenib for 2h, 4h, 8h, 20h, 24h. Western blots for SQSTM1 (62 kDa), LC3-I/II (~14-16 kDa), and GAPDH (~37 kDa)

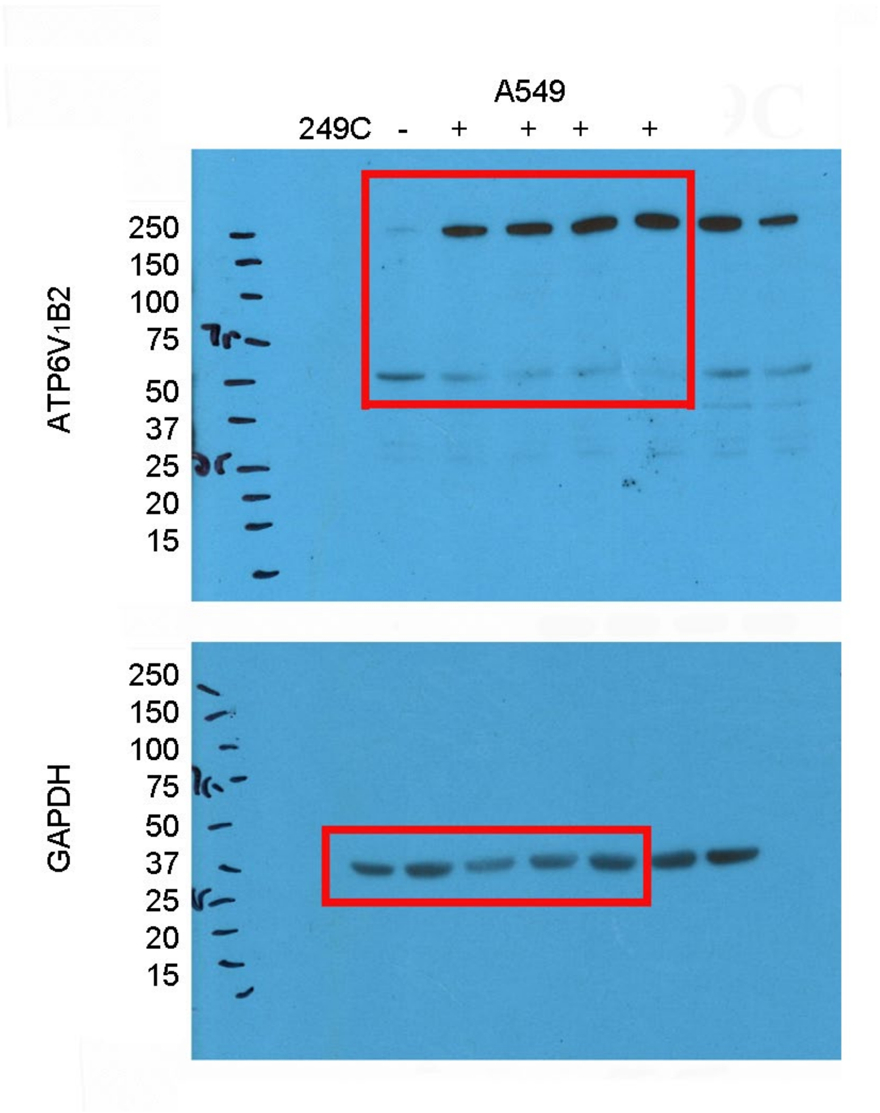

Supplementary Figure 15: Full-length Western blots for Supplementary Fig 5a. A549 cells were left untreated (DMSO) or treated with 249C for 2h, 4h, 8h, 20h, 24h. Western blots for $ATP6V_1B2$ (~54 kDa) and GAPDH (~37 kDa).

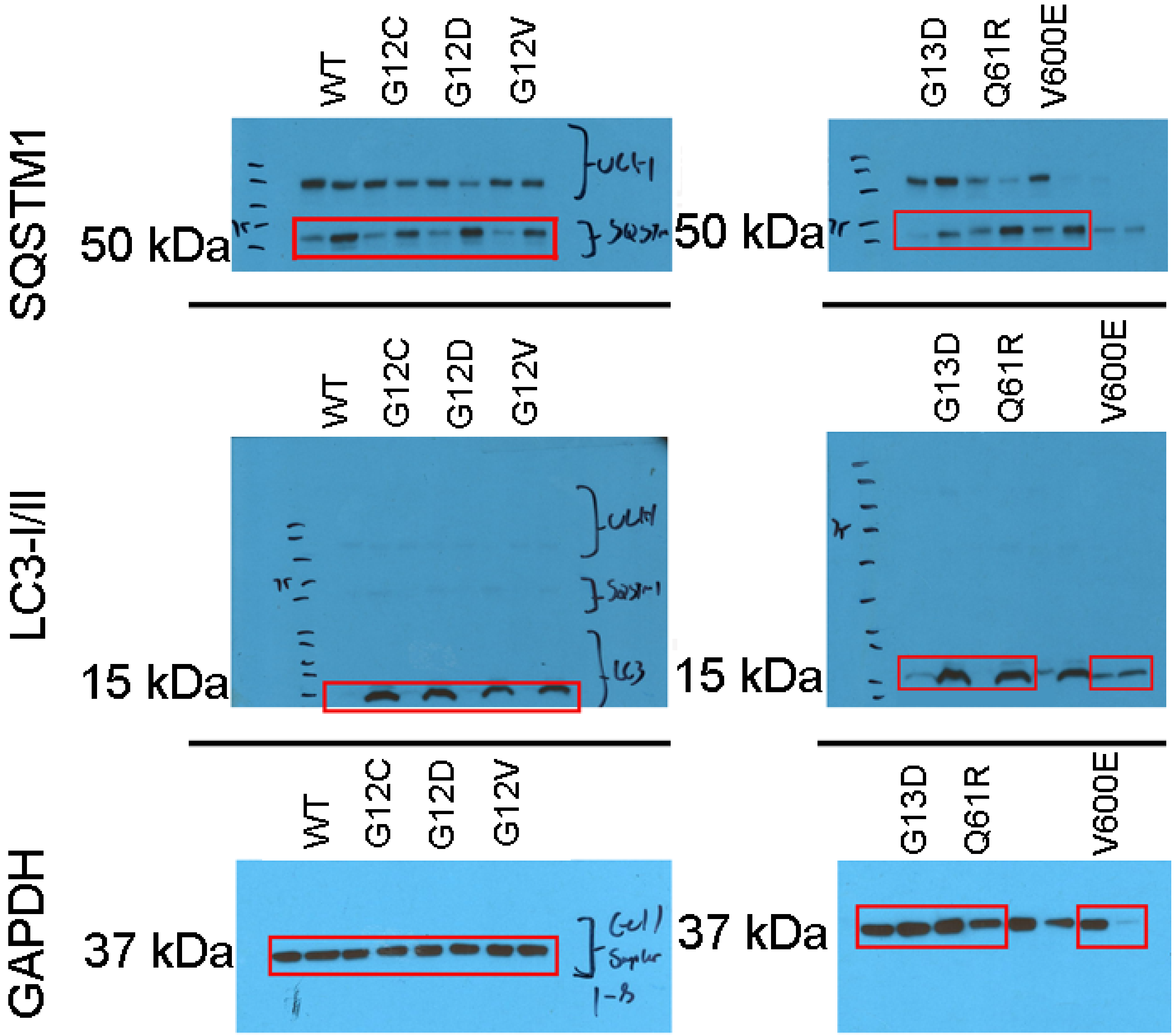

Supplementary Figure 16: Full-length Western blots for Supplementary Fig 7. MEF cells (WT, G12C, G12D, G13D, Q61R, V600E) were treated with DMSO or 249C (alternating). Western blot analysis for SQSTM1 (62 kDa), LC3-I/II (~14-16 kDa), and GAPDH (~37 kDa).

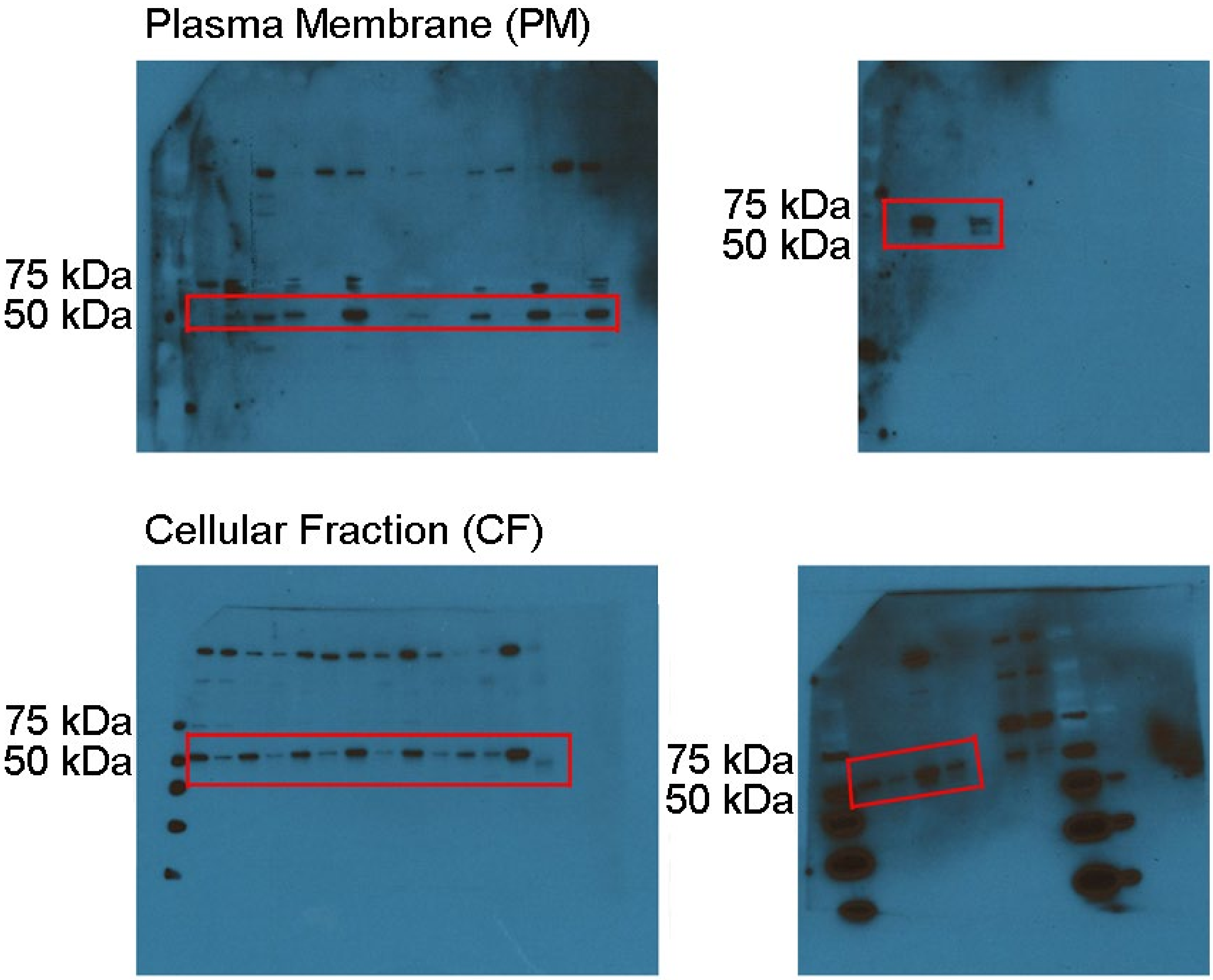

Supplementary Figure 17: Full-length Western blots for Supplementary Fig 8. Immunoblots of $ATP6V_1H$ (50-57 kDa) from the plasma membrane (PM) and cytoplasmic fraction (CF) of MEFs.

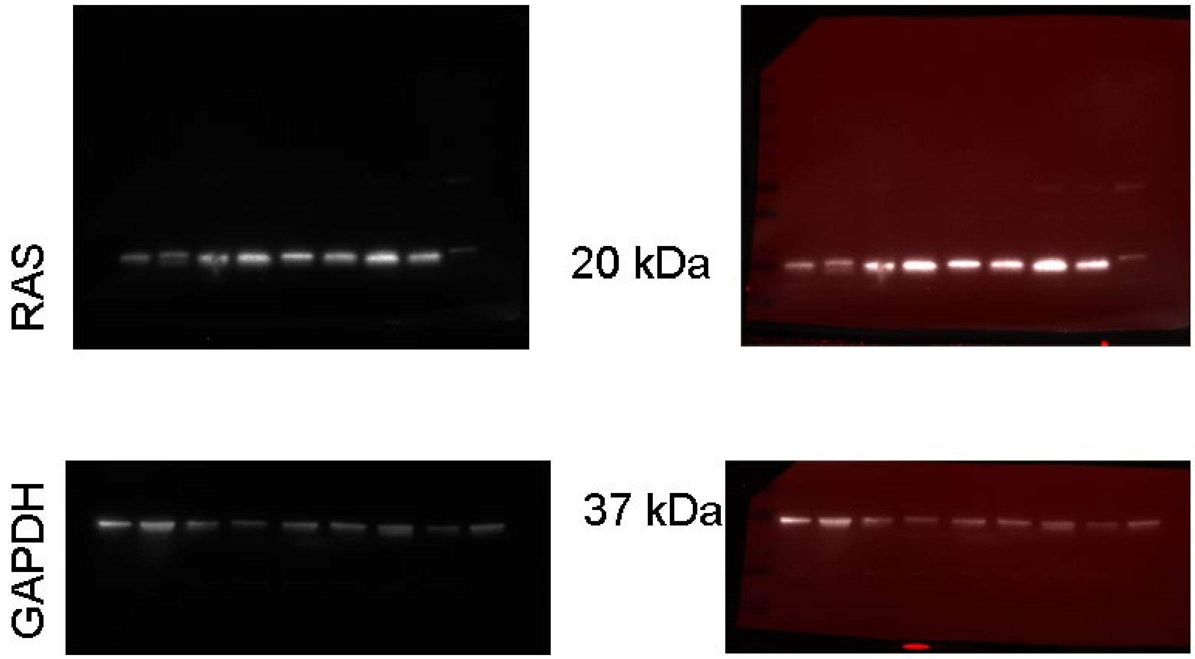

Supplementary Figure 18: Full-length Western blots for Supplementary Fig 8. MEF cells obtained from the NCI (*KRAS*WT, G12C, G12D, G12S, G12V, G13D, Q61L, Q61R and *BRAF*V600E) immunoblotted for Ras (~21 kDa) and GAPDH (~37 kDa), and imaged using a digital scanner.

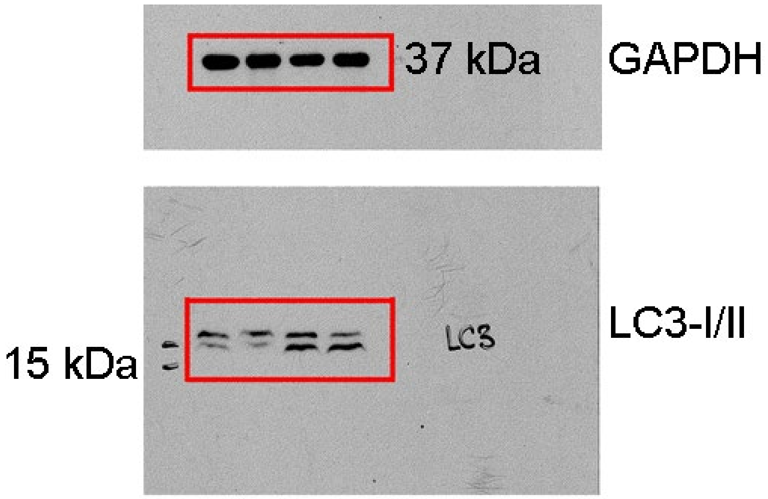

Supplementary Figure 19: Full-length Western blots for Supplementary Fig 11. *Ex vivo* analyses of 249C-treated tumors show an up-regulation of LC3-I/II expression when compared with the DMSO controls in A549 lung cancer xenografts. Lanes 1 + 2 = DMSO, 3 + 4 = 249C
