# Supplementary material for: Ras-mutant cancers are sensitive to small molecule inhibition of V-type ATPases in mice
Source: Nat Biotechnol. 2022 Jul 25;40(12):1834–44. doi: 10.1038/s41587-022-01386-z (PMC9750872; doi:10.1038/s41587-022-01386-z)
Supplement: Supplementary file 2 [file 41587_2022_1386_MOESM2_ESM.pdf]

Corresponding author(s): Bhairavi Tolani, Ph.D.
Last updated by author(s): May 8, 2022

Reporting Summary

Nature Portfolio wishes to improve the reproducibility of the work that we publish. This form provides structure for consistency and transparency in reporting. For further information on Nature Portfolio policies, see our Editorial Policies and the Editorial Policy Checklist.

Statistics

For all statistical analyses, confirm that the following items are present in the figure legend, table legend, main text, or Methods section.

n/a	Confirmed	
☐	☒	The exact sample size (n) for each experimental group/condition, given as a discrete number and unit of measurement
☐	☒	A statement on whether measurements were taken from distinct samples or whether the same sample was measured repeatedly
☐	☒	The statistical test(s) used AND whether they are one- or two-sided<br>*Only common tests should be described solely by name; describe more complex techniques in the Methods section.*
☒	☐	A description of all covariates tested
☒	☐	A description of any assumptions or corrections, such as tests of normality and adjustment for multiple comparisons
☐	☒	A full description of the statistical parameters including central tendency (e.g. means) or other basic estimates (e.g. regression coefficient) AND variation (e.g. standard deviation) or associated estimates of uncertainty (e.g. confidence intervals)
☐	☒	For null hypothesis testing, the test statistic (e.g. F, t, r) with confidence intervals, effect sizes, degrees of freedom and P value noted<br>*Give P values as exact values whenever suitable.*
☒	☐	For Bayesian analysis, information on the choice of priors and Markov chain Monte Carlo settings
☒	☐	For hierarchical and complex designs, identification of the appropriate level for tests and full reporting of outcomes
☐	☒	Estimates of effect sizes (e.g. Cohen's d, Pearson's r), indicating how they were calculated

Our web collection on statistics for biologists contains articles on many of the points above.

Software and code

Policy information about availability of computer code

Data collection

Cell Proliferation Assays: Data was collected using Promega's GLOMAX software (version 1.9.3)

Mutation Analysis in CCLE Cell Lines: Data from cell proliferation assays were used

Electron Microscopy: images were collected using Gatan Microscopy Suite DigitalMicrograph (version 3.4.3) and SerialEM (version 3.8.6)

Fluorescence Microscopy (LysoTracker, LysoSensor, ATP6V staining): Data was collected using ZEN blue from Carl Zeiss (2012)

CRISPR Screen: Next-generation sequencing data were collected using commercially available software from Illumina (HiSeq Software Suite v3.4.0)

Flow Cytometry: Data was collected FACSDiva software (version 8.0)

Proton Pumping: fluorescence data was collected using Gen5 (version 3.05) software from BioTek

Bio-Layer Interferometry Studies: Octet R software (version 12) was used for data collection

Macropinosome Visualization: Data were captured using Perkin Elmer Harmony® PhenoLOGIC™ software (version 3.2)

The following data were collected externally for a fee: Whole Proteome Mass Spectrometry, comparative profiling in a panel of primary cell-based systems, hERG activity, BioMAP Screening, in vivo plasma concentration, and the KINOMEscanTM screening.

Data analysis

Cell Proliferation Assays: Data was analyzed using Excel (2016) and GraphPad Prism (version 6)

Mutation Analysis in CCLE Cell Lines: Statistical analyses were performed in R (version 3.6.3). Multiple two-sided Wilcoxon rank sum tests were performed in R using the "wilcox.test" and "lapply" functions. The Combined Annotation Dependent Database (https://cadd.gs.washington.edu/) was used to assess the predicted functional importance of SNVs (version 1.6). Cell line mutation data (CCLE_DepMap_18q3_maf_20180718.txt) were downloaded from the Cancer Cell Line Encyclopedia (CCLE) at https://depmap.org/portal/download/ (2018).

Whole Proteome Analysis: GSEA software (version 4.2.3) and Molecular Signature Database (MSigDB version 7.4)

Electron Microscopy: images were montaged using TrakEM2 within Fiji (ImageJ version 1.53c). The montages were analyzed using IMOD (version 4.12.3). Data was analyzed with Excel and GraphPad Prism

Fluorescence Microscopy: No analyses were performed

CRISPR Screen: Sequencing reads from pooled CRISPR screens were aligned to the library sequences, counted, and quantified using the Python-based ScreenProcessing pipeline (https://github.com/mhorlbeck/ScreenProcessing). All analyses of screen data were performed in python3.7 using custom code based on Numpy (v1.15.4), Pandas (v0.25.3), and Scipy (v1.4.1). The p-values are derived from a two-sided Mann-Whitney U-test.

Flow Cytometry: Data was analyzed using Excel (2016)

Biochemical V-ATPase Assay and Proton Pumping: Commercially available software was used (Excel 2016 and GraphPad Prism v6)

Bio-Layer Interferometry Studies: Octet R software (version 12) was used for data analysis

Macropinosome Visualization: Data were analyzed using Perkin Elmer Harmony® PhenoLOGIC™ software (version 3.2)

3D Molecular Modeling, Binding Pocket Detection and Docking: All the software used is commercially available and described in the Methods section (EMBOSS Stretcher on EMBL-EBI webserver, DISSPRED webserver, MODELLER25 version 9.19, DOPE, Small Molecule Drug Discovery Suite 2019-127 (Schrödinger, LLC), SiteMap algorithm, Maestro v11.9, LigPrep v4.9, and Glide v.8.2)

The following data were analyzed externally: Whole Proteome Mass Spectrometry, comparative profiling in a panel of primary cell-based systems, hERG activity, BioMAP Screening, in vivo plasma concentration, and the KINOMEscanTM screening.

For manuscripts utilizing custom algorithms or software that are central to the research but not yet described in published literature, software must be made available to editors and reviewers. We strongly encourage code deposition in a community repository (e.g. GitHub). See the Nature Portfolio guidelines for submitting code & software for further information.

Data

Policy information about availability of data

All manuscripts must include a data availability statement. This statement should provide the following information, where applicable:

- Accession codes, unique identifiers, or web links for publicly available datasets
- A description of any restrictions on data availability
- For clinical datasets or third party data, please ensure that the statement adheres to our policy

Datasets that support the findings of this study are available in Source Data/Supplementary Figures and Tables.

Mutation Analysis in CCLE Cell Lines: The Combined Annotation Dependent Database (https://cadd.gs.washington.edu/) was used to assess the predicted functional importance of SNVs (version 1.6). Cell line mutation data (CCLE_DepMap_18q3_maf_20180718.txt) were downloaded from the Cancer Cell Line Encyclopedia (CCLE) at https://depmap.org/portal/download/ (2018).

Mass Spectrometry GO gene sets: GSEA (version 4.2.3) software and Molecular Signature Database (MSigDB version 7.4) from http://www.broad.mit.edu/gsea was used to query the top proteins from the Whole Proteome Analysis for GO gene sets in NCI-60 cell lines

CRISPR Screen: sgRNA read counts and phenotypes for all pooled screens are provided as supplementary tables

Field-specific reporting

Please select the one below that is the best fit for your research. If you are not sure, read the appropriate sections before making your selection.

☒ Life sciences ☐ Behavioural & social sciences ☐ Ecological, evolutionary & environmental sciences

For a reference copy of the document with all sections, see nature.com/documents/nr-reporting-summary-flat.pdf

Life sciences study design

All studies must disclose on these points even when the disclosure is negative.

Sample size	For all experiments, no sample size calculation was performed. Sample sizes were chosen empirically based on previously published work, knowledge of intra-group variation and expected effect sizes, or based on discussions with experts in their respective fields. These sample sizes were sufficient for statistical analysis.

Cell Proliferation Assays: Each compound OR cell line was tested a minimum of 2 times with n = 3

Mutation Analysis in CCLE Cell Lines: We analyzed mutations in the 53 cell lines that were also included in our MTS assays

Electron Microscopy: Images were taken from single replicate samples for each condition. A minimum of 10 cells for each sample were randomly imaged and measured for the scored parameters. For some structures, up to a total of 100 measurements were from the minimum of 10 cells for each condition.

CRISPR Screen: No sample size calculation was performed. The pooled genetic screen was performed as a single replicate. Each gene was targeted with 5 independent sgRNAs, providing internal replicates. Individual hits were validated in duplicate or triplicate in individual re-tests.

Flow Cytometry: Experiments were performed in duplicates at least

Biochemical V-ATPase Assay and Proton Pumping: at minimum, each bar represents mean +/- SD, n=3

Bio-Layer Interferometry Studies: Two individual experiments were performed

Macropinosome Visualization: For each cell line, 5 DMSO control and 5 249C-treated wells were imaged and a minimum of 3 images were captured per well

Animal Studies: sample size was decided based on discussions with the UCSF animal core facility

Data exclusions

CRISPR Screen: For the pooled screens, we required that sgRNAs had >49 counts in at least one of the two conditions that was being compared to calculate a given phenotype (pre-established exclusion criterion).

For all other experiments, no data were excluded.

Replication

All experiments were performed in duplicate or triplicate unless otherwise stated. All attempts at replication were successful. For the CRISPR screens, selected hits were replicated in duplicate or triplicate. For the electron microscopy, a minimum of 10 cells was captured for each sample in a single experiment. The biochemical V-ATPase assay was performed twice from one enzymatic preparation due to limitations on bovine tissue acquisition. Animal studies were performed once with 5 mice per treatment group housed separately.

Randomization

Animal Studies: Once the mean tumor reached a certain volume (between 100-200 mm3), animals were randomized into different treatment groups.

For all other experiments, cells or samples were randomly assigned to control or treatment groups.

Blinding

Electron Microscopy: The individuals who collected and analyzed data was blinded to group allocation during data collection and for data analysis.

Macropinosome Visualization: Fields of view were randomly selected using the Harmony software prior to automated imaging of the whole plate to ensure unbiased image acquisition and individuals who collected data were blinded to group allocation during data analysis.

Animal Studies: Individuals who performed the SW48 xenograft experiments were blinded to group allocation during data collection and for data analysis.

For all other experiments, blinding is not applicable as the quantitative analyses performed are not subject to human bias

Reporting for specific materials, systems and methods

We require information from authors about some types of materials, experimental systems and methods used in many studies. Here, indicate whether each material, system or method listed is relevant to your study. If you are not sure if a list item applies to your research, read the appropriate section before selecting a response.

Materials & experimental systems

n/a	Involved in the study	
☐	☒	Antibodies
☐	☒	Eukaryotic cell lines
☒	☐	Palaeontology and archaeology
☐	☒	Animals and other organisms
☒	☐	Human research participants
☒	☐	Clinical data
☒	☐	Dual use research of concern

Methods

n/a	Involved in the study	
☒	☐	ChIP-seq
☐	☒	Flow cytometry
☒	☐	MRI-based neuroimaging

Antibodies

Antibodies used

1) SQSTM1 (Cell Signaling: 8025, D5E2, Lot: 5)
2) LC3A/B (Cell Signaling: 12741T, (D3U4C) XP®, Lot: 4)

3) GAPDH (ThermoFisher: AM4300, 6C5, lot: 00439919)
4) ATP6V1B2 (ThermoFisher: PA5-52518, No clone#, lot: VJ3104174) + Received from Dr. Dennis Brown
5) ATP6V0D1/P39 (Abcam: ab56441, 2G12, lot: GR3299463-I)
6) ATP6V1H (Fisher: PIPA554793, No clone#, lot: UL289506)
7) ATP6V1A (Abnova: H00000523-M02, 4F5, lot: K5261) + Received from Dr. Dennis Brown
8) RAS (Cell Signaling: 3965, No clone#, lot: 4)
9) EGFR (Cell Signaling: 4267, No clone#, lot: 24)
10) Alexa-Fluor 488 goat anti-mouse IgG (ThermoFisher: A11001, No clone#, lot: 745480)
11) Alexa-Fluor 488 goat anti-rabbit IgG (ThermoFisher: A11008, No clone#, lot: 2179202)

Validation

All vendors showed validation data for antibodies on their websites (including Western blot, IF, IHC etc.)

Manufacturer's Website:
1) SQSTM1 (Cited in 195 publications: https://www.cellsignal.com/products/primary-antibodies/sqstm1-p62-d5e2-rabbit-mab/8025?site-search-type=Products&N=4294956287&Ntt=8025st&fromPage=plp)
2) LC3A/B (Cited in 521 publications: https://www.cellsignal.com/products/primary-antibodies/lc3a-b-d3u4c-xp-rabbit-mab/12741)
3) GAPDH (Cited in 474 publications: https://www.thermofisher.com/antibody/product/GAPDH-Antibody-clone-6C5-Monoclonal/AM4300)
4) ATP6V1B2 (Citations not listed; testing methodology provided: https://assets.thermofisher.com/TFS-Assets/LSG/Flyers/commitment-antibody-performance-flyer.pdf; validation provided on website: https://www.thermofisher.com/antibody/product/ATP6V1B2-Antibody-Polyclonal/PA5-52518)
5) ATP6V0D1/P39 (Cited in 18 publications: https://www.abcam.com/atp6v0d1p39-antibody-2g12-ab56441.html)
6) ATP6V1H (Citations not listed; validation provided on website: https://www.fishersci.com/shop/products/v-atpase-h-rabbit-anti-human-polyclonal-thermo-scientific-pierce/PIPA554793)
7) ATP6V1A (Cited in 2 publications including 1 in Nature: http://www.abnova.com/products/products_detail.asp?catalog_id=H00000523-M02)
8) RAS (Cited in 106 publications: https://www.cellsignal.com/products/primary-antibodies/ras-antibody/3965)
9) EGFR (Cited in 936 publications: https://www.cellsignal.com/products/primary-antibodies/egf-receptor-d38b1-xp-rabbit-mab/4267)
10) Alexa-Fluor 488 goat anti-mouse (Cited in 5438 publications: https://www.thermofisher.com/order/genome-database/dataSheetPdf?producttype=antibody&productsubtype=antibody_secondary&productId=A-11001&version=122)
11) Alexa-Fluor 488 goat anti-rabbit (Cited in 6210 publications: https://www.thermofisher.com/antibody/product/Goat-anti-Rabbit-IgG-H-L-Cross-Adsorbed-Secondary-Antibody-Polyclonal/A-11008)

Eukaryotic cell lines

Policy information about cell lines

Cell line source(s)

-MEF cell lines were obtained from the National Cancer Institute (NCI).
-SW48, SW48 KRASG12D/+, SW48 KRASG12V/+ and SW48 KRASG13D/+ cell lines were obtained from Horizon Discovery.
-MDA-MB-231 CRISPRi cells were obtained from Dr. Hani Goodarzi at UCSF. Parental MDA-MB-231 cells were from ATCC.
-Cell lines were obtained from ATCC: H1944 (CRL-5907), A427 (HTB-53), A549 (CRM-CCL-185), SW1271 (CRL-2177), H292 (CRL-1848), H358 (CRL-5807), H1299 (CRL-5803), H460 (HTB-177), H1703 (CRL-5889), H2170 (CRL-5928), H2452 (CRL-5946), NCI H82 (HTB-175), Calu-3 (HTB-55), H838 (CRL-5844), H1975 (CRL-5908), H1650 (CRL-5883), H28 (CRL-5820), CFPAC-1 (CRL-1918), Capan-1 (HTB-79), Panc 02-13 (CRL-2554), ASPC1 (CRL-1682), Panc1 (CRL-1469), BxPC3 (CRL-1687), MDA-MB-231 (HTB-26), Sk-Br-3 (HTB-30), MCF7 (HTB-22), MDA-MB-436 (HTB-130), HCT116 (CRL-247EMT), Colo201 (CCL-224), N87 (CRL-5822), RKO (CRL-2577), SW48 (CCL-231), CaCO2 (HTB-37), SkMel5 (HTB-70), SKMEL30 (HTB-73), A375 (CRL-1619), A2058 (CRL-11147), DUI145 (HTB-81), PC3 (CRL-3471), U251 (HTB-17), U87 (HTB-14), HL-60 (CCL-240), HepG2 (HB-8065).
LOX IMVI, MelJuso, BT474 and HCC1937 cells were a gift from the Oritz-Urda lab at UCSF originally sourced from ATCC or as gifts. ES2, OVCAR8, HEYA8, and HEY cells were a gift from the Smith-McCune lab at UCSF originally sourced from ATCC.

Authentication

-MEF parental cells were validated at the NCI using karyotyping. For the derived MEFs with KRAS mutations, the endogenous KRAS removal was authenticated via PCR to compare to a mouse reference gene Tfrc. MEF cells with subsequent addition of KRAS mutants were exome sequenced and compared with a mouse reference genome at a genomics lab (GRCm38 mm10).
-SW48 cells were validated by Horizon Discovery using genotyping (gDNA and cDNA):
https://horizondiscovery.com/-/media/Files/Horizon/resources/Data%20Sheets/88/HD%20103-011%2000058_DataSheet.pdf
https://horizondiscovery.com/-/media/Files/Horizon/resources/Data%20Sheets/31/HD%20103-007%2000053_DataSheet.pdf
https://horizondiscovery.com/-/media/Files/Horizon/resources/Data%20Sheets/70/HD%20103-002%2011917_DataSheet.pdf

-MDA-MB-231 CRISPRi cells were validated by Dr. Hani Goodarzi's lab.
-A549 cells were validated by ATCC using STR cell authentication services.
-Other cells obtained from ATCC relied on the company's certificates of analysis. No other independent authentication was performed.

Mycoplasma contamination

-MEF cells were tested for mycoplasma at the NCI and tested negative.
-Horizon Discovery states that all their cell lines are tested for mycoplasma in their Data Sheets.
-Dr. Goodarzi's lab tested for mycoplasma in MDA-MB-231 CRISPRi cells every 4 months.
-Our lab uses MycoAlert Detection Kits from Lonza Bioscience. All cell lines were tested for mycoplasma and were found to be negative.

Commonly misidentified lines
(See ICLAC register)

No misidentified cell lines that we know were used for this study.

Animals and other organisms

Policy information about studies involving animals; ARRIVE guidelines recommended for reporting animal research

Laboratory animals	-For A549 xenografts: 6-8-week old athymic nude female mice (Charles River strain # 088, homozygous NU/NU)<br>-For SW48 xenografts: 6-8-week old athymic nude female mice (Taconic model # NCRNU-F, CrTac:NCR-Foxn1 sp/sp)
Wild animals	The study did not involve wild animals.
Field-collected samples	The study did not involve samples collected from the field.
Ethics oversight	All animal procedures were performed under IACUC approved protocols and guidelines. Mouse manipulations were performed in accordance with UCSF's IACUC protocol AN179973.

Note that full information on the approval of the study protocol must also be provided in the manuscript.

Flow Cytometry

Plots

Confirm that:

☒ The axis labels state the marker and fluorochrome used (e.g. CD4-FITC).

☒ The axis scales are clearly visible. Include numbers along axes only for bottom left plot of group (a 'group' is an analysis of identical markers).

☐ All plots are contour plots with outliers or pseudocolor plots.

☐ A numerical value for number of cells or percentage (with statistics) is provided.

Methodology

Sample preparation	-For CRISPR re-test experiments, populations of cells containing 20-50% sgRNA-expressing cells were treated with the indicated compounds for the indicated times, dissociated, and immediately run on the flow cytometer without further processing.<br><br>-For Apoptosis Assays: MEFs from the NCI or cancer cells obtained from ATCC were treated with 100 nM 249C for 48 hours and following two washes with ice-cold PBS, they were stained using a FITC Annexin V/PI Apoptosis Detection kit (BD Pharmingen) by following the manufacturer's recommendations.
Instrument	BD™ LSR II flow cytometer (Serial# H47100133) - BD Biosciences
Software	FACSDiva software (version 8.0) was used for data acquisition and Excel (2016) was used for data analyses.
Cell population abundance	For Apoptosis Assays: Samples were analyzed by flow cytometry but no cell sorting was performed.
Gating strategy	-For CRISPR re-test experiments, an FSC/SSC gate was applied followed by a BFP gate to demarcate sgRNA-expressing cells.<br><br>-For Apoptosis Assays: Preliminary FSC/SSC gates were applied followed by removal of doublets (FSC-H vs FSC-A and also SSC-H vs SSC-A). Next single color controls (FITC only or PI only) and double positive controls (FITC+ & PI+) were run to set gates for the double positive population. Finally, untreated and 249C-treated samples were run keeping these gates constant for all.

☒ Tick this box to confirm that a figure exemplifying the gating strategy is provided in the Supplementary Information.
